# Artificial Intelligence in the Diagnosis of Superficial Mycoses: A Scoping Review

**DOI:** 10.3390/jof12090684

**Published:** 2026-09-11

**Authors:** Denisa Mihaela Loghin (Misăiloaie), Ioana Adriana Popescu, Dan Vâță, Mădălina Mocanu, Laura Gheucă-Solovăstru

**Affiliations:** 1 Department of Dermatology, “Grigore T. Popa” University of Medicine and Pharmacy, 700115 Iași, Romania; 2 Department of Dermatology, “St. Spiridon” Emergency County Clinical Hospital, 700111 Iași, Romania

**Keywords:** artificial intelligence, deep learning, superficial mycoses, onychomycosis, dermatophytosis, diagnostic accuracy, scoping review, external validation

## Abstract

Artificial intelligence (AI) is increasingly proposed for diagnosing superficial mycoses, yet whether this literature covers the diagnostic pathway evenly, and is mature enough for clinical translation, remains unclear. We mapped AI applications onto an operational partition of the diagnostic pathway (D1–D4), a construct of this review: pre-analytical clinician-facing imaging (D1), analytical microscopy and histopathology (D2), analytical molecular identification (D3) and integrative post-analytical interpretation (D4). Thirty-three primary studies (2007 to 2026) were included, 85% published since 2021 and predominantly from Asia. Applications concentrated on D2 (*n* = 16) and D1 (*n* = 13), with few in D3 (*n* = 4) and none in D4. Reported accuracy was frequently high but rested on internal, single-centre validation: complete external validation and prospective preregistration were each present in a single study, only a handful shared both public code and public data, about half reported no demographic data and no D1 study stratified performance by Fitzpatrick phototype. A substantial minority carried conflicts of interest tied to the evaluated tool, concentrated in the molecular domain where independent verification was scarcest; where such re-evaluation was possible, reported accuracy fell markedly (one area under the curve (AUC) from 0.98 to 0.75). This corpus is skewed toward the early diagnostic steps, leaves the integrative post-analytical step unaddressed and shows methodological maturity insufficient for routine clinical translation. Institutionalised external validation, reproducibility through open code and data, declaration of dataset overlap and conflicts of interest, and clinically meaningful outcomes are the priorities for future work.

## 1. Introduction

*“It makes no sense to perform clinical research without ensuring clinical utility.”*—John P. A. Ioannidis, *Why Most Clinical Research Is Not Useful* (2016).

[1]

Artificial intelligence (AI) is increasingly proposed for the diagnosis of superficial fungal infections. Like clinical research more broadly, these tools will ultimately be judged by their real-world clinical utility [1]; the obstacles to that utility have recently been grouped into four areas: model accuracy, regulation and ethics, legal implications and access [2]. This review therefore asks where AI applications sit within the diagnostic pathway of superficial mycoses and how far their methodological maturity supports clinical use.

Such maturity cannot be assumed. In a review of AI for COVID-19, only 62 of 2212 studies had adequate methodology and none proved clinically usable, owing to limitations in validity, validation and reporting [3]. The benchmark against which performance is claimed can itself be unstable: a convolutional neural network (CNN) compared with 145 dermatologists on 100 images faced extreme inter-rater variability (sensitivity 55–100%, specificity 22.5–92.5%) [4], so “dermatologist-level” claims warrant caution. Two principles are therefore central to reading this literature critically, each a point at which reported performance may be inflated: transfer learning, the reuse of a model pretrained on a large general dataset and adapted to a small medical one, whose features may not transfer well to medical images [5,6]; and stochastic analysis, the repeated training of a model under different random initialisations to report a distribution rather than a single, possibly unrepresentative, run [4].

Briefly, on terminology: AI aims to reproduce cognitive functions such as learning and pattern recognition; within it, machine learning (ML) improves from examples without explicit programming, and deep learning (DL), a subset of ML, uses multi-layered neural networks that learn features directly from raw data [5,7]. CNNs, inspired by the visual cortex, remain the most common DL architecture for medical image analysis [5] and account for most of the applications reviewed here, although the corpus also spans classical ML, transformer-based models and ML on molecular signals.

Against this methodological background stands the clinical problem. Superficial fungal infections affect an estimated 20–25% of the global population, rising to about 60% in some tropical regions [8,9], and are among the most common problems in primary care, with US costs estimated at about USD 845 million in 2019 [9]. Yet diagnosis is difficult. Clinical examination is accessible but error-prone: among board-certified dermatologists, accuracy fell below 75% in 9 of 13 cases [10]. Atypical presentations are a persistent source of misdiagnosis: tinea faciei mimicking seborrhoeic dermatitis or discoid lupus, moccasin-type tinea pedis mistaken for eczema or psoriasis, tinea manuum misread as contact dermatitis [11,12] and tinea incognito from corticosteroid-antifungal self-medication [8]. Empiric therapy without mycological confirmation exposes patients to adverse effects and can foster resistance [13], a concern underscored by terbinafine resistance in *Trichophyton rubrum* and *T. interdigitale* [14] and, notably, in *T. indotineae*, which carries squalene epoxidase (SQLE)-mediated resistance in over half of strains [8].

Laboratory confirmation, though treated as the reference standard, is itself imperfect. In onychomycosis, a meta-analysis of 2858 subjects found pooled sensitivities of only 61% for potassium hydroxide (KOH) microscopy, 56% for culture and 84% for periodic acid-Schiff (PAS) nail histopathology, with high specificities but extreme inter-study heterogeneity [15]. Each method has characteristic weaknesses: KOH is operator-dependent [16]; culture is slow, its apparent specificity inflated by contaminants, and species identification depends on an experienced mycologist [17,18]; molecular assays (internal transcribed spacer [ITS] PCR-RFLP, or microarrays) are rapid and species-specific but largely confined to specialist centres and uneven in coverage [19,20], although a mixed calcofluor-plus-PCR algorithm reached 98.2% sensitivity and 100% specificity against a composite standard [17]. The methods also differ in what they establish: direct microscopy shows fungal presence but not species, identification relies on culture morphology and often on sequencing [21] and PAS histopathology is a workflow distinct from direct microscopy [16]. Proof-of-concept AI at this step, such as a Vision Transformer detecting Malassezia in pityriasis versicolor (AUC-ROC 0.99) [22] and a telemycology study of digital images [18], remains monocentric and externally unvalidated. Experience with AI in dermatology, furthermore, has accumulated mainly in oncology, where the task is a binary malignant-versus-benign classification of pigmented lesions on dermoscopy; this transfers only partly to mycology, where the useful question is multi-class and therapy-linked (which species, whether resistant and what treatment), as a systematic review of AI in onychomycosis also notes [23].

These difficulties share a common root: the diagnosis of superficial mycoses is not a single act but a multi-step pathway, from the clinician–patient encounter through direct microscopy, culture and molecular identification to the interpretation that turns a laboratory result into a treatment decision. AI can enter this pathway at any of these steps, yet each step differs in its input, its user and the decision it serves, so the value of a given tool (together with the obstacles it must still clear, from accuracy to regulation, legal implications and access [2]) depends on where in the pathway it sits. Judging clinical utility [1] therefore requires more than a headline accuracy figure; it requires knowing which step of the diagnostic process an application serves, and how methodologically mature it is at that step.

### Objectives of This Review

This scoping review examines the current literature on the application of artificial intelligence (AI) to the diagnosis of superficial mycoses, pursuing three complementary objectives:

Mapping the technologies and diagnostic tasks reported in the literature, ranging from lesion classification on clinical photography and dermoscopy to the analysis of direct microscopy images (potassium hydroxide [KOH], calcofluor white, tape strip), histopathology (periodic acid-Schiff [PAS], Grocott methenamine silver [GMS], haematoxylin and eosin [H&E]) and molecular substrates (matrix-assisted laser desorption/ionisation-time of flight [MALDI-TOF] mass spectrometry, internal transcribed spacer [ITS] sequencing and microarray).

Positioning the identified AI applications along the diagnostic pathway of superficial mycoses, using an operational grid (D1–D4) that we define by three attributes of each application: its input, its intended user and its output. The grid is a working construct of this review, not a validated taxonomy. Its laboratory steps (analytical and post-analytical, D2–D4) are instantiated from the French ANOFEL/LABAC recommendations for the laboratory diagnosis of superficial mycoses [21], whereas the initial clinician-facing step (D1) is anchored in clinical practice guidelines rather than in that laboratory document. This positioning allows coverage and gaps to be identified at each step: the clinician–patient encounter (D1), analytical detection by microscopy or histopathology (D2) and by molecular techniques (D3) and integrative post-analytical interpretation (D4).

Identifying the methodological gaps and the directions required for AI applications in this field to become translatable into real-world practice, with emphasis on the internal and external validity of the studies analysed.

The central question is not whether the included studies are methodologically impeccable, but whether the current AI literature on superficial mycoses covers the steps of the diagnostic pathway in a balanced manner, and whether the methodological maturity of the corpus is sufficient to support transfer into practice. The following section details the literature selection criteria, the D1–D4 operational grid and the descriptive conventions used to evaluate each application domain.

## 2. Materials and Methods

### 2.1. Approach and Reporting Standard

This review was conducted as a scoping review with narrative elements, following the Joanna Briggs Institute (JBI) guidance (Table 1—PCC framework) [24] and reported in accordance with PRISMA-ScR (Preferred Reporting Items for Systematic Reviews and Meta-Analyses extension for Scoping Reviews) [25], with the flow-diagram structure adapted from PRISMA 2020 [26]. Unlike a systematic review, the aim was not the quantitative synthesis of individual results but the mapping of the literature landscape and the descriptive appraisal of the clinical utility of the identified AI applications. This distinction follows from the analytic framework set out in Section 2.4.

The protocol was not preregistered. This absence is acknowledged as a methodological limitation and discussed in the Limitations section.

### 2.2. Eligibility Criteria (PCC Framework)

Eligibility was established using the Population-Concept-Context (PCC) framework recommended by the JBI for scoping reviews [24]:

**Table 1:** PCC framework.

	Inclusion Criteria	Exclusion Criteria
Population	Human patients with suspected or confirmed superficial mycoses: dermatophytoses (all forms of tinea), cutaneous candidiasis, pityriasis versicolor and onychomycosis.	All animal-model studies were excluded, irrespective of pathogen type or imaging modality. This reflects the review’s stated aim, clinical utility in human practice, and ensures consistency of the appraisal criteria.
Concept	Any artificial intelligence application (classical machine learning, deep learning, foundation models, vision-language models [27]) intended for diagnosis or identification of the causative pathogen.	Studies dedicated exclusively to treatment, drug discovery, pharmacological prognosis or epidemiological modelling were excluded.
Context	Primary care, outpatient dermatology, the clinical laboratory and telemedicine. Patient-facing applications (mobile apps for self-screening) were included only if they had been evaluated in a real clinical setting with real patients; in this approach, the effective user of the application remains the clinician who interprets the AI output.	Studies whose data came exclusively from non-clinical sources (internet images such as Google Images or Bing, public image databases such as DermNet or ISIC, or synthetic images), with no validation cohort of real patients in a clinical setting, were excluded.

Data source. To be eligible, a study had to include at least one cohort of real human patients in a clinical context. Studies based exclusively on datasets assembled from internet images (for example, Google Images or Bing), public dermatological image databases (for example, DermNet or ISIC), synthetic images or other non-clinical data were excluded. The use of public data for training or augmentation was acceptable provided the study also included a validation cohort of real patients. The presence of physicians, dermatologists or laboratory technicians as human assessors or comparators was not in itself an inclusion criterion: studies with human participants in a comparator role were eligible only if the imaging or laboratory data originated from real patients, and the methodological value of a real human comparator is recorded descriptively in the Results section (internal-validity subcategory).

Eligible publication types were peer-reviewed original articles, book chapters, conference papers with a complete methodology and preprints indexed in major repositories (arXiv, bioRxiv, medRxiv). Editorials, commentaries, conference abstracts without a methodology and articles of a metaphorical or educational nature lacking primary data were excluded. Only English-language publications were considered. No date restriction was applied: earlier studies, including those predating the widespread adoption of deep learning, were retained to document the historical trajectory of the field.

### 2.3. Information Sources and Search Strategy

Five complementary databases were queried on 7 May 2026, the date that defines the corpus cutoff: PubMed/MEDLINE and Europe PMC for the peer-reviewed biomedical literature, and arXiv, OpenAlex and Dimensions for the computer-science and conference literature that forms a substantial part of AI research but is underrepresented in biomedical databases. The search combined conjunctive blocks of artificial-intelligence terms and superficial-mycoses terms, using Medical Subject Headings (MeSH) and text-word variants, where supported, and adapting the syntax to each platform. All searches were limited to English, with no restriction on publication year. The master search (PubMed/MEDLINE) was as follows:

(“Artificial Intelligence”[MeSH] OR “Deep Learning”[MeSH] OR “Machine Learning”[MeSH]

 OR “Neural Networks, Computer”[MeSH] OR “convolutional neural network*”[tiab] OR “deep learning”[tiab] OR “machine learning”[tiab] OR “computer-aided diagnos*”[tiab] OR “computer assisted diagnos*”[tiab] OR “image analysis”[tiab] OR “vision transformer*”[tiab])

AND

(“Tinea”[MeSH] OR “Onychomycosis”[MeSH] OR “Dermatomycoses”[MeSH] OR “Candidiasis, Cutaneous”[MeSH] OR “Tinea Versicolor”[MeSH] OR dermatophytos*[tiab] OR “superficial myco*”[tiab] OR “fungal skin infection*”[tiab] OR “nail myco*”[tiab] OR onychomycos*[tiab] OR tinea[tiab] OR “pityriasis versicolor”[tiab] OR “cutaneous candid*”[tiab])

AND

(“Diagnosis”[MeSH] OR “Diagnostic Imaging”[MeSH] OR diagnos*[tiab] OR detect*[tiab]

 OR classif*[tiab] OR identif*[tiab])

Filters: Humans; English; no date restriction.

**Figure 1:**
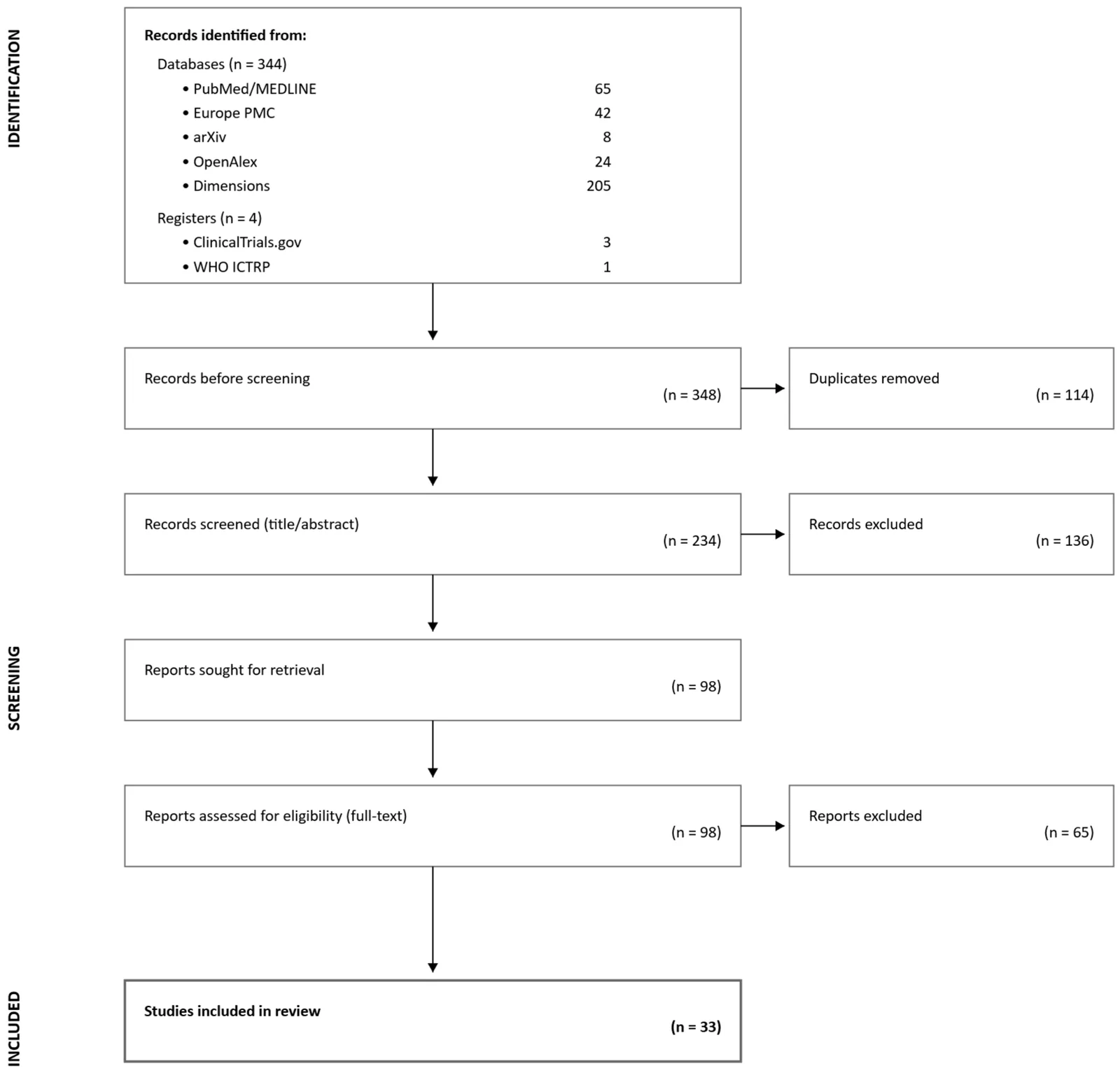
PRISMA-ScR flow diagram.

For Europe PMC, arXiv, OpenAlex and Dimensions, the two-pillar structure (artificial intelligence AND superficial mycoses) was adapted to each platform’s syntax; the complete search string for every database is given in Supplementary Materials Table S4. On arXiv, where the combined Boolean query returned no records, disease names were searched as successive single terms with manual screening. Result counts per source are reported in the PRISMA-ScR flow diagram (Figure 1).

Following PRISMA-ScR, complementary sources for primary studies comprised trial registries (ClinicalTrials.gov, WHO ICTRP) and backward citation chasing, both on the included studies and on the reference lists of the comparator reviews. The comparator reviews themselves were assembled separately from the primary-study search and do not form part of it: they were identified pragmatically, from the database searches, from supplementary searching on Google Scholar and from the reference lists and citations of other reviews and were used to position the present contribution. This set is purposive and is not intended to be exhaustive or fully reproducible; it does not enter the PRISMA-ScR flow diagram and is separate from the 33 primary studies. Scopus and Web of Science were not queried. Forward citation chasing (screening the studies that cite an included study) was likewise not performed; it is not mandatory for scoping reviews under PRISMA-ScR [25] or the JBI manual [24], and the Cochrane Handbook [28] lists it only as a method that “should be considered” for systematic reviews. Both omissions are acknowledged in the Limitations section.

### 2.4. Selection Process

Screening was carried out in two successive rounds: (a) title-and-abstract triage, followed by (b) full-text appraisal of the articles that passed the first round. The exclusion criteria applied at each level are those specified in Section 2.2. The flow of records through identification, screening and inclusion is summarised in the PRISMA-ScR flow diagram (Figure 1).

Single reviewer. Both the title-and-abstract triage and the full-text appraisal were performed by a single reviewer. The JBI/PRISMA-ScR standard recommends the use of two independent reviewers, followed by resolution of disagreements through discussion or by the intervention of a third reviewer. The absence of a second independent reviewer is a methodological limitation explicitly acknowledged in the Limitations section; its potential effects include a risk of erroneous exclusion of relevant studies (false negatives at triage) and of inclusion of studies that do not strictly meet the PCC criteria.

#### 2.4.1. Unit of Analysis: The AI Application

The unit of analysis in this review is the AI application, not the individual study. By “AI application”, we mean a combination of three elements:

the input (clinical photograph, dermoscopic image, microscopic field, MALDI-TOF spectrum, ITS sequence, microarray, etc.);

the intended user (dermatologist, family physician, microbiologist, pathologist, reference laboratory);

the output (diagnostic probability, fungal-element detection, species identification, integrated decision support, etc.).

We chose these three elements because, in our view, they help place an application within the diagnostic pathway according to the step it serves, rather than by the technology used or the disease targeted, which were the criteria on which most of the earlier classifications examined in Section 3 were organised. For this reason, we consider it more appropriate that a study should not be judged useful or useless in itself, but in relation to the application it serves: for example, five studies with different CNN architectures for identifying hyphae on KOH preparations are treated as a single application, same input, same user, same output, evaluated in five technically distinct ways.

These three elements form what we call the D1–D4 operational grid (Table 2): it places an application on the step of the diagnostic pathway it serves, not on the technology and not on the pathology. That pathway is not a national particularity but the universal structure of the total testing process (pre-analytical, analytical, post-analytical), the sequence codified for medical laboratories in ISO 15189; within it, the analytical and post-analytical steps (D2–D4) are operationalised in detail from the French ANOFEL/LABAC recommendations, while the clinician-facing step (D1) draws on clinical practice guidelines rather than that laboratory document. The D1–D4 partition proposed below is a construct of this synthesis, not a term borrowed from the cited source, and is intended as a working grid rather than a validated taxonomy; we acknowledge that other allocations are possible, and these are discussed in the Limitations section. Where the same step serves different substrates or users, we chose to subdivide explicitly (for example, direct microscopy, histopathology and culture reading within the visual analytical step), so as not to imply a homogeneity that these sub-domains do not have.

Absence of the sampling step among the grid domains. The diagnostic pathway includes a pre-analytical laboratory step, site selection, the specimen collection itself, transport and assessment of specimen quality, performed by trained personnel [21]. This step does not appear as a domain of the D1–D4 grid for a reason related to the unit of analysis: the grid classifies AI applications by input, user and output, and no application in the examined corpus takes specimen collection as its object; to our knowledge, there are also no published AI applications dedicated to this step. Sampling is therefore not a populated domain but a real step of the pathway that AI applications do not address; we flag it explicitly as a coverage gap, not as an omitted element. This distinction also clarifies the boundary of the D1 domain: clinical-image analysis occupies only the pre-diagnostic moment of the clinical decision, not the entire pre-analytical phase, whose collection and conditioning steps remain outside what image-oriented applications can serve.

#### 2.4.2. D1—Clinical, Pre-Diagnostic Step

D1 sits at the clinician–patient interface, before any specimen exists: the clinician assesses the lesion and decides whether, and what, to investigate further. It includes clinical photography and dermoscopy on the basis of their function, and clinical imaging before sampling. Two boundaries apply. D1 is not the pre-analytical laboratory phase: the sampling steps come after the clinical decision and are not addressed by image-based applications. Even with several images per patient, D1 is a narrower input than the full clinical examination, which includes history and the inspection of other sites; D1 applications therefore support the clinician decision rather than replace it. Patient-facing self-screening without medical supervision is not appraised, as the intended user is the clinician who interprets the output.

#### 2.4.3. D2—Analytical, Visual Substrate

D2 covers the visual component of the analytical laboratory phase: the reading of an optical substrate for fungal elements. Direct examination and culture are the first analytical steps in the ANOFEL/LABAC recommendations [21], and the same visual methods appear in recent Canadian reviews [29,30]. It groups three sub-domains that share the output (visual detection of the fungal element) but differ in input and user, and whose metrics are not combined because their reference standards differ: D2a, direct microscopy of fresh material (KOH, calcofluor or blankophor fluorescence, native tape strip for *Malassezia*; anchored in direct examination [15,16,21]); D2b, histopathology of fixed tissue (PAS, GMS, H&E, whole-slide imaging), which does not appear in the ANOFEL/LABAC laboratory document and is anchored instead in the broader diagnostic literature [15,16,30] and included by functional homology; and D2c, culture reading (colony morphology) [21]. D2 stops at visual detection or identification of the element; species identification from a molecular signal is D3, even on the same culture.

#### 2.4.4. D3—Analytical, Molecular Substrate

D3 covers the molecular component of the analytical phase, where the input is a raw molecular signal: mass spectrometry [21], ITS/TEF1 sequencing [21], terbinafine-susceptibility testing [21] and multiplex PCR [21]; the same methods appear in the Canadian reviews [29,30]. Its therapeutic relevance stems from emerging terbinafine resistance, in particular, *T. indotineae* [8]. Because the user and output are constant across platforms (MALDI spectrum, sequence, microarray), D3 is not subdivided. It begins where the input is molecular, visual colony reading being D2c, and stops at identification and the resistance prediction derived from that signal; the move to therapy is D4.

#### 2.4.5. D4—Integrative, Post-Analytical Step

D4 covers the post-analytical phase, where the raw result becomes interpretation and action, closing the total testing loop [31]. In the ANOFEL/LABAC recommendations, this is the interpretation of the result, establishing pathogenicity and distinguishing colonisation from infection [21], and the biologiste-conseil role [21]. Its input is not a raw substrate but the laboratory result integrated with the clinical picture and epidemiological context, so it differs from the earlier steps in all three attributes at once. Because a domain defined by integration could seem hard to populate, we separate a universal core (an interpretive report or a resistance or atypical-pathogenicity alert, issuable by a laboratory professional in most systems) from a jurisdiction-dependent extension (a direct therapeutic recommendation, which in the French model falls to the biologiste-conseil [21] but elsewhere may not). Coverage is reported against this minimal, internationally shared core, so weak coverage cannot be attributed to an artificially restrictive definition.

### 2.5. Data Synthesis

The synthesis is narrative, organised around the four application domains. For each populated domain, the Results give a short description of the domain and the types of studies addressing it, a synthesis of the comparable quantitative results (sensitivity, specificity, AUC) and a narrative appraisal of the descriptive methodological features. Per-study characteristics (design, dataset size, population, modality, architecture, principal metrics) are summarised (one row per study across the corpus) and given in full in Supplementary Materials Tables S1–S3. The review articles are used as context for positioning the present contribution.

#### Reporting Conventions

Confirmed N. A study has a “confirmed N” only if it states the number of distinct real human patients in the evaluation cohort; studies reporting only images, slides, patches, specimens, isolates or colonies are recorded as “N = NR (not reported)”, even when that unit is objective.

Setting. Each study is assigned to one category (primary care/secondary hospital/tertiary hospital/referral laboratory/community/NR); a free-text description is mapped to this taxonomy in the Setting column.

External validation. We record it as an ordinal descriptive summary of three levels, complete, partial and none (shown as “Yes”, “Partial”, “No”), used to characterise the corpus and not as a claim that studies within a level are methodologically equivalent. The “partial” level in particular groups designs that are not equivalent, for example, a single-centre application of an externally developed algorithm and a temporal (same-institution) split; the specific design for each study is therefore stated in the cell note and in Supplementary Materials Table S3 rather than being collapsed into the label.

Human comparator. The presence of a real human comparator is recorded descriptively, with number and qualifications.

Metric reporting. A study reporting only sensitivity and specificity, without at least one predictive value or likelihood ratio, is flagged as having minimal reporting.

Same-substrate expert reference. In some studies, the AI is evaluated against a human reading of the same substrate that the model itself processes (for example, human KOH microscopy used as the reference standard for an AI operating on KOH images). This is a same-substrate expert reference, which is not in itself circular; its limitation is narrower: because the index test and the reference share the same substrate and its intrinsic error, the AI can at best reproduce, not exceed, that reading, so the configuration caps the demonstrable ceiling of performance rather than invalidating the comparison. It is recorded descriptively as a limitation of internal validity rather than an exclusion criterion: the studies concerned are retained in the corpus and flagged in the Results (Section 4.2).

Conflicts of interest. Industry ties were documented descriptively, using an operational, non-validated convention rather than a formal risk-of-bias instrument. Four categories record the degree of industry involvement: maximal (an author is a founder or employee of the company that produces the evaluated tool), high (two or more industrially affiliated authors, none a founder), moderate (a single industrially affiliated author) and low (a kit or reagent supplied without the company’s participation in study design). The categories summarise disclosed relationships and are not a judgement of study validity; the underlying relationships are reported per study in Supplementary Materials Tables S3 and S5.

### 2.6. Descriptive Methodological Framework

This is a scoping review, so a formal risk-of-bias assessment was not an objective and was not performed, an option PRISMA-ScR explicitly permits [25]. The four QUADAS-2 domains [32], patient selection, index test, reference standard and flow and timing, are used only as an organising taxonomy for the descriptive extraction of methodological features, with no risk-of-bias judgment at domain or study level. QUADAS-2 does not address features specific to AI applications (external validation across data distributions, architectural transparency, pipeline reproducibility) [33]; the updated QUADAS-3 [34] appeared close to the corpus cutoff and was not adopted, and the dedicated QUADAS-AI extension [33,35] was not yet available in consensus form when the corpus closed.

### 2.7. Use of AI-Assisted Tools

During the preparation of this review, the authors used an AI-assisted tool to support two defined tasks: (i) cross-checking the numerical and categorical data extracted from the included primary studies (sample sizes, performance metrics, reference standards, conflict-of-interest disclosures and study characteristics) against the original publications, to verify their accuracy and traceability to the source; and (ii) bibliography management, that is, the organisation and formatting of references that had already been identified, retrieved and verified by the authors. The tool was a private, non-public application, without a commercial name, that integrates six large language models and applies cross-checking between their outputs. The literature search, study screening and selection, and retrieval of full-text articles were performed entirely by the authors; the AI tool was not used to search for, identify, select or retrieve studies or references, nor to generate data or produce any scientific interpretation, analysis or conclusion. All AI-assisted outputs were reviewed and confirmed by the authors against the primary sources, and the authors take full responsibility for the entire content of the manuscript.

## 3. Background: Prior Review Literature on AI in Superficial Mycoses

The review literature on the application of artificial intelligence (AI) to the diagnosis of superficial mycoses has expanded markedly since 2020, driven by the parallel growth of dermatological AI research and clinical awareness of the diagnostic limitations of conventional mycology. This section positions the present work against that literature; it is a narrative comparison, not a second systematic review. It draws on the purposive set of comparator reviews described in Section 2.3 (*n* = 14; Table 3). An initial mapping of this set reveals a corpus heterogeneous in scope, depth and analytic framework, a heterogeneity that is itself informative for understanding where knowledge has consolidated and where it remains fragmentary.

### 3.1. Scope and Focus of the Existing Reviews

The existing reviews divide into two groups by primary focus. The first comprises reviews dedicated explicitly to AI in superficial mycoses or in nail pathology: Bulińska et al. [23], Hasan Pour [36], Lim et al. [37], Gaurav et al. [38], Shakir & Mahdi [39] and Durdu & Ilkit [40]. The second comprises reviews in which superficial mycoses are one of several conditions discussed: seven address AI in general dermatology (Hogarty et al. [41], De et al. [42], Biswas et al. [43], Ye & Chen [44], Della Mura et al. [45], Gupta & Hall [29] and Gupta et al. [30]), while Albuqami et al. [46] surveys AI for fungal infections across organ systems, with only two of its eleven included studies concerning superficial mycoses.

In the first group, the focus is predominantly, and in several cases exclusively, on onychomycosis. Bulińska et al. [23] is the most methodologically rigorous contribution, a systematic review applying QUADAS-2 that included 14 manuscripts (11 primary AI studies and 3 reviews), across clinical photographs, dermoscopic images, digitised histopathology (whole-slide imaging of PAS) and KOH microscopy. Lim et al. [37] offered a narrative synthesis of AI applied to nail diagnosis, positioning it within a broader algorithmic framework that integrates conventional methods, dermoscopy and AI. Gaurav et al. [38] extended this coverage to the whole spectrum of nail pathology, of which onychomycosis is the best-documented AI application. This near-exclusive concentration on onychomycosis reflects the structure of the primary literature itself: nail images offer a coherent, morphologically accessible substrate, and the diagnostic challenge, distinguishing onychomycosis from inflammatory nail disorders, maps naturally onto binary classification. Hasan Pour [36] synthesised 54 studies across three domains (diagnosis, treatment, prevention), of which 19 concern diagnosis, and noted that AI research on non-nail dermatophytoses remains comparatively scarce. Durdu & Ilkit [40], although a clinical review of dermatophytosis management, integrates AI as one element of the diagnostic toolkit alongside microscopy, dermoscopy and molecular methods, a positioning close to the framework of the present review. Shakir & Mahdi [39] reviewed deep-learning methods (CNN, Transformer and hybrid architectures) for skin fungal infection detection, summarising about 26 studies by algorithm, dataset and reported limitations, and highlighting data diversity and explainability as the main barriers to clinical adoption, without a structured synthesis of performance.

Albuqami et al. [46] belongs to this group by a different route: rather than surveying dermatology broadly, it surveys AI for fungal infection across organ systems (ocular, respiratory, ENT, dermatological and molecular), within which two onychomycosis studies fall inside the superficial-mycoses scope; a further study, on cutaneous cryptococcosis and talaromycosis, addresses deep mycoses with cutaneous involvement and lies outside that scope. It is nonetheless the only review in the corpus to address the triage function of AI explicitly, and the only one in which no included study addresses dermatophyte species identification or antifungal-resistance prediction despite covering AI for fungal species identification in other systems.

Among the reviews addressing general dermatology, coverage of superficial mycoses is consistently marginal. Hogarty et al. [41], De et al. [42] and Ye & Chen [44] each mention onychomycosis as a proof of concept for AI in infectious dermatology, typically citing Han et al. [47] as the anchor study, without extending the analysis to other forms of superficial mycosis. Biswas et al. [43] similarly restrict their fungal coverage to onychomycosis, drawing on general-purpose fungal and multi-disorder AI work rather than on a dedicated onychomycosis benchmark. Della Mura et al. [45] stand out for including a section on AI in the dermatopathology of cutaneous fungal infections alongside inflammatory conditions, identifying histopathological detection of fungal elements as an insufficiently explored application, with only a single study identified to date; the analysis, however, remains descriptive and limited to a small subset of studies. Gupta & Hall [29] and Gupta et al. [30] provide comprehensive syntheses of the diagnostic methods for onychomycosis (AI included) in the context of a broad review of conventional and emerging technologies; their value for the present work lies in their critical appraisal of diagnostic-accuracy metrics and in their explicit recognition that sensitivity and specificity figures must be interpreted against the limitations of the reference standard itself.

**Table 3:** Synthesis of the review literature on AI in superficial mycoses and dermatology (*n* = 14), in chronological order.

Reference	Y	Design	Scope	Mycoses Coverage	AI Studies	AI Domains	Clinical Utility Discussed
Hogarty et al. [41]	2019	NR	Dermatology—AI	Onychomycosis	1	D1	Overdiagnosis concerns
De et al. [42]	2020	NR	Dermatology—AI	Onychomycosis	1	D1	None
Lim et al. [37]	2021	NR	Onychomycosis—diagnosis	Onychomycosis	2	D1, D2	Integration into a diagnostic algorithm proposed
Gupta & Hall [29]	2022	NR	Onychomycosis—diagnostic methods	Onychomycosis only	5	D1, D2	Yes
Gupta et al. [30]	2022	NR	Onychomycosis—diagnostic methods	Onychomycosis	5	D1, D2	Yes—AI is in early stages
Ye & Chen [44]	2023	NR	Dermatology—AI	Onychomycosis (mentioned)	2	D1	None
Durdu & Ilkit [40]	2023	NR	Dermatophytosis—diagnosis and management	Dermatophytosis only	NR	NR	Partial—AI within the diagnostic workflow
Bulińska et al. [23]	2024	SR	Onycomycoses—AI	Onychomycosis	11	D1, D2	Partial—performance vs. clinicians; no workflow integration
Shakir & Mahdi [39]	2024	NR	Cutaneous fungal infections—AI	Onychomycosis, tinea, candidiasis	26	D1, D2	Minimal—implementation not evaluated
Biswas et al. [43]	2025	NR	Dermatology—AI	Cutaneous fungal infections	2	D1	None
Della Mura et al. [45]	2025	NR	Dermatopathology—AI	Cutaneous fungal infections, onychomycosis	2	D2	Minimal—performance metrics only
Gaurav et al. [38]	2025	NR	Nail pathology—AI	Onychomycosis (+other nail disorders)	10	D1, D2	Minimal—clinical-adoption barriers listed
Hasan Pour [36]	2025	NR	Superficial mycoses—AI	Onychomycosis, dermatophytosis, candidiasis, PV	19	D1, D2, D3	Partial—AI as adjunct noted; no structured evaluation
Albuqami [46]	2026	SR	Fungal infections (all systems)—AI vs. conventional methods	onychomycosis ×2	2	D1	AI for triage

AI domains: D1 = clinical/dermoscopic image analysis; D2 = microscopy/histopathology; D3 = species identification/antifungal resistance. PV = pityriasis versicolor; NR = not reported; SR = Systematic review; NR = Narrative review.

### 3.2. Shared Findings and Common Limitations

Across these reviews, a few observations recur, though with varying rigour. For onychomycosis, CNNs trained on clinical, dermoscopic or microscopic images tend to perform on a par with, and occasionally above, dermatologists, with internally validated AUCs commonly around 0.82 to 0.98 (first reported by Han et al. [47] and repeated in several later reviews [23,29,30,37,38,40]). For KOH microscopy, AI classifiers have reported accuracies of roughly 88 to 95%, exceeding the average dermatologist on accuracy and specificity though not consistently on sensitivity (Yilmaz et al. [48]; also noted in [23,39,40]). In histopathology, AI on PAS-stained sections has been reported as non-inferior to dermatopathologists in two studies, Decroos et al. [49] and Jansen et al. [50], both cited by Bulińska [23] and Gaurav [38], with Decroos also discussed by Della Mura [45]. These observations recur across reviews of differing rigour, from narrative syntheses to the QUADAS-2-filtered analysis of Bulińska [23]; that breadth lends them some weight, but they rest largely on internally validated datasets and should be read with that limitation in mind.

The reviews that offer critical, rather than descriptive, analysis point to a similar set of limitations: small, single-centre datasets with limited ethnic diversity; a preference for internal over external or prospective validation; binary classification that does not capture the multiclass nature of real diagnosis, noted by Bulińska et al. [23]; and limited calibration reporting. No unified public benchmark for superficial mycoses yet exists, and Shakir & Mahdi [39] point to sparse explainable AI (XAI) reporting as a further barrier to clinical use.

### 3.3. What the Existing Reviews Do Not Address

The reviews acknowledge structural limitations, but none maps AI applications onto the diagnostic pathway of superficial mycoses. Performance is benchmarked against statistical reference standards (culture, histopathology, KOH positivity) rather than the decisions clinicians or laboratory professionals make at each step, so the practical value of, say, an AUC-0.92 dermoscopic triage tool for general practitioners, or an 88%–accurate KOH classifier for reducing unwarranted empirical antifungals, is rarely examined. Two features of the primary literature are carried in uncritically: research concentrates on the clinical or dermoscopic image, the dermatologist’s entry point, leaving the laboratory pathway (microscopy, culture, molecular characterisation) comparatively neglected despite its role as the technical reference standard; and AI for species identification and antifungal-resistance prediction, the tasks with the most direct therapeutic weight, is largely absent despite the urgency of terbinafine-resistant *Trichophyton indotineae* [36,40].

This review addresses that gap with an operational grid (D1–D4), a construct of this synthesis rather than a pre-existing scheme: its laboratory steps (D2–D4) draw on the French ANOFEL/LABAC recommendations [21], and its clinician-facing step (D1) on European (BAD [51], Nenoff et al. [52], Mayser et al. [53]), North American (Lipner & Scher [16]) and Canadian (Gupta & Hall [29], Gupta et al. [30]) guidelines and reviews, applied to the 33 included primary studies.

## 4. Results

### 4.1. General Characteristics of the Included Studies

The included studies are summarised in Table 4 (one row per study, grouped by application domain; D1–D3 populated, D4 empty), together with their general, internal and external-validity characteristics; full per-study data are provided in Supplementary Materials Tables S1–S3. The corpus spans successive generations of AI methods, from pre-deep-learning systems through convolutional networks and Vision Transformers to machine learning applied to molecular signals. Geographically, the studies originate predominantly from Asia and Europe, with a smaller number from North America (all from the United States) and Africa, and none from Latin America or Oceania. Methodological concerns, including dataset overlaps, conflicts of interest and specific design limitations, are noted in the Comments column of Supplementary Materials Tables S1–S3.

The corpus comprises 33 primary studies published between 2007 and 2026, the large majority of them (29 of 33, 88%) since 2021, and originates predominantly from Asian centres, notably China, South Korea, India, Thailand and Japan. Under the review’s D1–D4 grid, the distribution across steps is uneven: 16 studies fall in D2, 13 in D1, 4 in D3 and none in D4. This pattern mirrors the availability of accessible imaging data and leaves the decision-integration step under-represented.

Reported performance is generally high but heterogeneous and inconsistently documented: many studies report accuracy or sensitivity and specificity, rather than AUC, and where AUC is available, it ranges from about 0.75 to 0.99, with the highest values resting on internal or single-centre validation. Safeguards against overoptimistic estimates are, by contrast, uncommon: complete external validation, prospective preregistration and the joint availability of public code and data were each reported in only a small minority of studies, demographic reporting was frequently absent and several studies carried competing-interest or dataset-overlap concerns. These features are documented per study in Section 4.2 and Section 4.3 and in Supplementary Materials Tables S1–S3 and S5; taken together, they raise the possibility that reported performance is systematically overestimated.

The corpus is also technologically heterogeneous, with four generations of methods coexisting. Pre-deep-learning approaches are represented by Berger [54] (PAPNET;Neuromedical Systems, Inc., Suffern, NY, USA) and Mäder [55] (rule-based processing using Canny edge detection, morphology and deterministic classification), published eight years apart and included for thematic relevance. Classical machine learning is exemplified by Duzayak [56] (artificial neural network, support vector machine and ensemble of decision trees), explicitly motivated by clinical hardware constraints. Convolutional neural networks predominate across D1 and D2 (ResNet, VGG, DenseNet, EfficientNet and YOLO), while more recent architectures include U-Net variants with residual and attention modules, the convolutional-Transformer hybrid of Kong [57], the multi-stage pipeline of Miura [58] and pure Vision Transformers (Köberle [22], ViT-B/32; Yodrabum [59], Swin Transformer; and, in part, Miura [58], ViT-B). Transitions between these generations are not clearly demarcated in the literature. The four D3 studies span both paradigms and use distinct molecular signals: microarray [60,61], MALDI-TOF [62] and ITS Sanger sequencing [63].

**Table 4:** Synthesis of the included studies (*n* = 33), by AI application domain. Full per-study data are provided in Supplementary Materials Tables S1–S3.

Study (Year, Country)	Modality/Substrate	Design	N	Reference Standard	Primary Metric (95% CI)	Human Comp.	External Validation
D1—Pre-analytical (clinician-facing image processing)—*n* = 13							
Duzayak [56] (2023, Turkey)	Clinical photos (nails)	Prospective consecutive	76	Specialist clinical dx (no KOH/culture)	AUC 0.97; Se 0.96; Sp 0.98	No	No
Han [47] (2018, S. Korea) *	Clinical photos	Retrospective; development + multi-site external validation	4557 (dev.) + 342 (external validation)	KOH/culture/antifungal response	AUC 0.98 → 0.82 (B1 → D)	Yes	Yes (3 independent sites)
Idress [64] (2025, Sudan)	Clinical photos	Prospective cross-sectional	350	KOH + culture	AUC 0.955; Se 95.5%; Sp 91.6%	Yes	No
Julanon [65] (2025, Thailand)	Clinical photos	Retrospective	580	KOH/histopathology/culture (tinea); > = 2/3 dermatologist consensus (non-tinea)	AUC 0.84 (0.80–0.88)	Yes	Partial (temporal)
Kim [66] (2020, S. Korea)	Dermoscopic images	Prospective (external test of Han2018c)	90	KOH 40% + culture	AUC 0.751 (0.646–0.856)	Yes	Partial (external algorithm)
Kong [57] (2025, China)	Clinical photos	Retrospective dev./val.	NR (1155 img)	>=2 dermatologist consensus + >=1 positive lab/histopathology test	AUC 0.877	No	No
Mehta [67] (2024, India)	Clinical photos	Prospective cross-sectional	257	≥2 board-cert. derm. consensus	AUC 0.99 (tinea cruris); 0.68 (candidiasis)	Yes	Partial (external algorithm)
Miura [58] (2024, Japan)	Clinical photos (foot)	Retrospective dev./val.	50	Expert clinical evaluation	Recall 0.907; Acc 0.897	No	No
Nigat [68] (2023, Ethiopia)	Clinical photos	Retrospective	NR (407 img)	Dermatological dx	Se 86.4%; Sp 95.4%	No	No
Pangti [69] (2021, India)	Clinical photos	Prospective cross-sectional	5014	≥2 board-cert. derm. consensus	AUC 0.95 (tinea cruris/corporis/faciei)	No	No
Police [70] (2025, India)	Smartphone clinical photos (3 apps)	Prospective cross-sectional	32 ( mycosis subset *n* = 8)	Board-cert. derm. consensus	Top-1 tinea corporis 50–75%	No	No
Yodrabum [59] (2026, Thailand)	Clinical photos	Mixed retro + prospective	NR (mixed)	KOH + derm. review	Precision 0.925; Recall 0.980 (dermatophytosis, Swin)	Yes	No
Zhu [71] (2022, China)	Dermoscopic images	Retrospective dev./val.	206	KOH and/or culture and/or PAS+	Youden 0.715 (vs. dermatologists 0.427–0.444)	Yes	No
D2—Analytical, visual substrate (microscopy/histopathology)—*n* = 16							
Berger [54] (2007, Netherlands)	Histopathology (PAS sections)	Interobserver reproducibility	NR (50 nail clippings)	Light microscopy	Kappa 0.70–0.97	Yes	No
Decroos [49] (2021, Germany)	Histopathology (PAS)	Retrospective DTA	199	2 dermatopathologists, consensus → 2 dermatopathologists (consensus); PCR for 49 uncertain negative cases	AUC 0.981; Se 94.1%; Sp 98.0%	Yes	No
Gagna [72] (2024, USA)	Histopathology	Descriptive-methodological	14	Conventional histopathology	Accuracy 82%	No	No
Gao [73] (2021, China)	Fluorescence microscopy	Prospective validation	292 specimens	Human microscopy (2 experts)	Se 96.8%; Sp 93.2%	Yes	No
He [74] (2025, China)	Fluorescence microscopy (FMIA)	Prospective DTA	300	Composite (clinical + mycology)	Se 96.3%; Sp 94.9%; AUC 0.96	Yes	No
Hulse [75] (2026, USA)	Histopathology (PAS), decision support	Retrospective cohort	64	PAS histochemistry	Best model: Firth logistic reg. (bootstrap CI)	Yes (4 assessors)	No—preregistered (OSF)
Jansen [50] (2022, Germany)	Histopathology (PAS WSI)	Retrospective, multicentre	NR (74 WSI val.)	11 dermatopathologists, consensus	Se 94%; Sp 77%	Yes	No
Koberle [22] (2024, Germany)	Tape-strip microscopy (glass slide)	Classifier dev./val.	NR (22 specimens)	Expert mycologist annotation	Se 94.0%; Sp 93.5%	No	No
Koo [76] (2021, S. Korea)	KOH microscopy	Retrospective object-detection	38	Human KOH microscopy (2 derm.)	Se 99.0%; Sp 86.6%	No	No
Mäder [55] (2015, Germany)	Microscopy	Algorithm dev./val.	NR (415 img)	Experienced clinician classification	Se 83%; Sp 79%	Yes	No
Rajitha [77] (2022, India)	KOH microscopy	Retrospective CNN dev.	NR (2626 patches)	Clinical classification	Se 97.6%; Sp 99.4%	No	No
Rajitha [78] (2025, India)	KOH microscopy	Semantic segmentation	NR (patches)	Manual segmentation masks	Dice 76.4–84.6	No	No
Rajitha [79] (2025, India)	KOH microscopy	Quantitative grading	NR (378 img)	2 microbiologists, manual grading	Fleiss kappa (auto vs. manual)	Yes	No
Rappoport [80] (2024, Israel)	Histopathology (PAS/GMS WSI)	Retrospective	NR (22 slides)	2 specialist pathologists (overall revision)	GMS: Se 0.93; Sp 0.99	No	No
Ren [81] (2025, China)	Fluorescence microscopy	Dual-model (development/validation)	NR (942 img)	2 clinicians (annotation, double-checked)	Recall 99.3%; Precision 92.5%	Yes	No
Yilmaz [48] (2022, Turkey)	KOH microscopy	Case-control accuracy + human comparison (16 dermatologists)	10 (7073 patches)	Human KOH microscopy (2 derm.)	Se 74.9%; Sp 93.8%; AUC 0.934	Yes	No
D3—Analytical, molecular substrate—*n* = 4							
Anton [60] (2023, France)	DNA microarray	Prospective-retrospective tech. validation	NR (284 specimens; 87 patients in swab group)	Culture (COFRAC); RT-PCR or NGS for discordant	Se 83–100%/Sp 99–100% (LOOCV, 441-sample training DB)	No	No
Ivagnes [62] (2025, Italy)	MALDI-TOF	Retrospective ML validation	NR (56 isolates)	ITS sequencing + ERG	Balanced accuracy 100%	No	No
Tirard-Collet [61] (2025, France)	PCR + microarray	Retrospective validation	NR (85 specimens)	Mycological culture → Mycological culture; pan-fungal PCR + Sanger sequencing (for discordant)	Se 83.9% (72.8–91.0); Sp 88.9% (95% CI 67.2–96.9)	No	Partial (manufacturer-independent)
Todd [63] (2026, USA)	ITS sequence (CNN genotyping)	Retrospective surveillance; CNN development/validation	188	Manual ITS alignment	CNN vs. manual concordance 98.7%	Yes	No
D4—Integrative post-analytical step—*n* = 0 (declared empty)							

Conventions: Patients (N) = distinct patients (dev. + test); NR = the study reports only images/patches/specimens/isolates/slides. External validation: Yes = independent multi-site; Partial = single-centre external algorithm or temporal split; No = internal only. Human comp. = real human comparator reported. * Han et al. datasets B1 and D. B1 and D are two of the external-validation datasets used by Han et al. [47], drawn from different institutions: B1 = Inje University (*n* = 100, culture-confirmed), on which the model reached AUC 0.98; D = Seoul National University (*n* = 939, KOH-confirmed), on which it reached AUC 0.82. The notation “0.98 → 0.82 (B1 → D)” in the Primary-metric column therefore records the drop in AUC on transfer between institutions.

### 4.2. Internal Validity of the Included Studies

Table 4 summarises several methodological features that bear on the internal validity of the included studies, namely, the reference standard, sample size, validation type, human comparator and the reported primary metrics with their confidence intervals where available; Supplementary Materials Table S2 provides further detail. Section Reporting Conventions defines the three operational grades of validation: complete external, partial external and internal only. Substantial heterogeneity in reference standards and reported primary metrics is observed across the corpus.

The presence or absence of a real human comparator was recorded for each study. Those with a robust human comparator, using multiple assessors stratified by experience or institution, include Han et al. [47] (42 dermatologists and 57 non-dermatologist assessors), Zhu et al. [71] (54 dermatologists), Jansen et al. [50] (11 dermatopathologists from 10 institutions) and Hulse et al. [75] (4 assessors ranging from student to dermatopathologist).

A same-substrate expert reference applies to three studies [48,55,76], in which the AI is evaluated against a human reading of the same substrate that it processes (for example, human KOH microscopy versus AI on KOH). This is not automatically circular, but performance metrics from these studies should still be interpreted with caution, because the index test and the reference share the same substrate and its intrinsic limitations, so the model can at best match, not exceed, that reading.

### 4.3. External Validity and Reproducibility

Several features relevant to external validity and reproducibility, which we do not treat as an exhaustive set, are summarised per study in Supplementary Materials Table S3 and quantified here: patient selection, demographic reporting, single- versus multi-centre design, protocol preregistration, degree of external validation, availability of code and data and conflicts of interest. The operational grading systems for external validation (three levels) and conflicts of interest (four levels) are defined in Methods.

Patient selection was prospective and consecutive in 12 studies, retrospective in 18, mixed in two [47,59] and purposive in one [70]. Twenty-seven studies (82%) were single-centre; three used independent multi-institutional data [47,50,62], and three combined several sources around a single reference centre or public repositories [59,65,69].

Complete demographic data (age, sex and relevant clinical characteristics) were reported in 10 studies (30%), partial data in eight (24%) and none in 15 (45%). No D1 study stratified performance by Fitzpatrick phototype.

Complete external validation across independent institutions was reported in one study (3%) [47] and internal validation only in 28 (85%). The four studies (12%) classed as partial [61,65,66,67] did not use a single, equivalent design, but rather a single-centre application of an externally developed algorithm or a temporal split; they are therefore itemised individually in Supplementary Materials Table S3 rather than treated as a uniform category.

Protocol preregistration was present in a single study (3%) [75].

Public code was available for four studies [22,47,63,65] and public data for four [47,63,75,76]; both code and data were available together in two (6%) [47,63]. Two studies explicitly stated that code was unavailable [78,81].

Dataset overlap. Undeclared dataset overlap across successive publications from the same group was identified in 5 of 33 studies (15%), in two clusters: three D2 publications by one research group [77,78,79] and a pair of D1 studies from a single institution [57,71] in which the training and test images overlapped. None of these studies declared the overlap in their Methods section.

Conflicts of interest were present in 10 of 33 studies. Maximal risk (an author is a founder or employee of the company producing the evaluated tool) was assigned to four [55,60,69,80], high risk (two or more industrially affiliated authors, none a founder) to three [49,50,81], moderate risk (a single affiliated author) to two [62,73] and low risk (a kit or reagent supplied without the company’s participation in study design) to one [61]. Excluding the single low-risk case, nine of 33 studies (27%) carried quantifiable risk beyond minimal. Several disclosures were incomplete: Rappoport et al. [80], Decroos et al. [49], Jansen et al. [50], Mäder et al. [55] and Gao et al. [73] declared no or no quantifiable conflict despite industrially affiliated co-authors or a proprietary reagent tied to the evaluated tool. Four studies carried no competing-interest statement at all—a 2007 letter and three conference papers [54,57,58,64]; the remaining 19 declared no conflict and showed no identifiable industrial affiliation. The full per-study conflict-of-interest information is provided in Supplementary Materials Table S5.

### 4.4. The D4 Category

The D4 category, which encompasses AI applications dedicated to the integrative post-analytical step, interpretive reporting, therapeutic recommendation, antifungal-resistance alerting and the biologiste-conseil role, is unpopulated in the present corpus (0 of 33 studies). This absence does not result from methodological omission or from the eligibility criteria; it reflects a structural gap in the current literature. At corpus closure, no published AI application dedicated to this step was identified.

Two studies show partial interfaces with D4, although neither addresses it as a primary objective. Hulse et al. [75] (D2) produces a decisional output on whether to order an additional PAS stain, and Todd et al. [63] (D3) establishes an implicit chain from the convolutional neural network genotype to the antifungal-susceptibility-testing (AFST) resistance profile to a therapeutic decision. The mechanisms and implications of these two transitional cases are examined in the Discussion.

## 5. Discussion

### 5.1. Findings

We identified 33 primary studies (2007–2026), 85% published since 2021 and predominantly from Asia. Mapped onto the D1–D4 partition, the corpus is unevenly distributed (D1 = 13, D2 = 16, D3 = 4, D4 = 0), concentrating on the image-rich early steps while leaving the integrative post-analytical step unaddressed, and it is technologically heterogeneous, spanning four coexisting generations from pre-deep-learning systems to Vision Transformers and machine learning on molecular signals.

Reported metrics are frequently high but the methodological foundation is thin. Discrimination was documented inconsistently, with the area under the ROC curve given in only 15 of 33 studies (range 0.75 to 0.99), and the highest figures rest on internal or single-centre validation, so a corpus-wide claim of high accuracy is not supportable.

Safeguards against optimistic estimates were rare: complete external validation and prospective preregistration were each present in a single study (3%) [47,75], both public code and data in two (6%) [47,63], demographic data were absent in 15 (45%) and no D1 study stratified performance by Fitzpatrick phototype, leaving performance across skin tones uncharacterised [82]. That reported accuracy may be systematically optimistic is shown by the two algorithms that degraded when re-evaluated on new populations, one AUC falling from 0.98 to 0.75 [47,66], consistent with an abundant AI literature that can fail to reach clinical usability [3].

The principal structural finding is the complete absence of AI dedicated to the integrative post-analytical step (D4); only two studies reach it partially and incidentally, Hulse et al. [75] (D2) and Todd et al. [63] (D3). In sum, the corpus is expanding rapidly but unevenly, strongest where imaging data are easiest to obtain and weakest, or absent, precisely where a laboratory result must become a clinical decision.

### 5.2. Contribution of the Project

The principal conceptual contribution is the D1–D4 partition, a construct of this review. Built on the universal structure of the total testing process (pre-analytical, analytical, post-analytical) [31], its laboratory steps (D2–D4) are instantiated from the French ANOFEL/LABAC recommendations [21] and its clinician-facing step (D1) is anchored in clinical guidelines from several jurisdictions (Section 3.3), so it is not tied to a single national scheme. Unlike taxonomies organised by technology (CNN, classical machine learning, Transformer) or by pathology (onychomycosis, tinea, pityriasis versicolor), it classifies each application jointly by user, input and the diagnostic step it serves. None of the 14 prior reviews maps applications to the steps of the pathway, and the empty D4 category is a direct product of this mapping: without an explicit integrative post-analytical step, the absence of AI for interpretive reporting and therapeutic recommendation is not visible as a structural gap.

A second contribution is to separate two patterns of data reuse that are easily conflated. The first, undeclared dataset overlap, is a methodological problem: five of 33 studies (15%) reuse images across successive same-institution publications without disclosure, and in one cluster, the training and test images themselves overlap, a form of data leakage that inflates performance and is a recognised driver of the reproducibility crisis [83] (examined in Section 5.4.1). The second, its desirable opposite, is re-evaluation of a published algorithm on a new population with explicit reporting of the performance change: Kim et al. [66] re-tested Han et al. [47] (N = 90; AUC 0.98 to 0.75), Mehta et al. [67] re-tested the DermaAId(Nurithm Labs Private Limited, Noida, India) tool of Pangti et al. [69] (N = 257; entity-differential degradation) and Tirard-Collet et al. [61] evaluated the platform of Anton et al. [60] (technical failure rate 5.9%). Only the last is institutionally independent, however, the other two sharing authors or an institution with the original report, so genuine outside verification remains rare even among these (examined further in Section 5.5.3).

### 5.3. Emergent Patterns by Domain

#### 5.3.1. D1—Pre-Analytical, Clinician-Facing

All thirteen D1 studies operate on clinical or dermoscopic images captured with widely accessible devices (smartphones, digital cameras, portable dermatoscopes), without laboratory equipment, which makes D1 the domain with the greatest potential for clinical scalability. Methodological rigour, however, is uneven, and only one study is a robust exemplar: Han [47] is the sole study in the corpus with complete external validation across three independent institutions (342 distinct patients), public code and data and transparent reporting of the performance decline on inter-institutional transfer (AUC 0.98 to 0.82). The others fall short: only 2 of 13 share public code, proprietary algorithms are common (DermaAId in Pangti [69] and Mehta [67]; the commercial apps Aysa (VisualDx, Rochester, NY, USA), Skinner (Arboreal Bioinnovations Pvt Ltd, India) and AI Dermatologist Skin Scanner (AI4SKIN, Toronto, ON, Canada) evaluated by Police [70]) and 9 of 13 report internal validation only. No D1 study stratifies performance by Fitzpatrick phototype, so performance on darker skin remains uncharacterised for these tools [82] (Section 5.4.2).

#### 5.3.2. D2—Analytical, Microscopy and Histopathology

D2 is the most heterogeneous domain, both quantitatively (16 studies, the largest per domain) and qualitatively. Its technological span is wide: Berger [54] (PAPNET, a scanning-based neural network, 2007) and Mäder et al. [55] (rule-based processing with Canny edge detection, morphology and deterministic classification, 2015) sit alongside more recent methods such as Decroos [49] (a VGG-13-like architecture with formal statistical validation), Köberle [22] (a Vision Transformer, ViT-B/32, on native tape strip for *Malassezia*) and Rajitha [78] (a U-Net with residual and attention modules for segmentation). This range reflects the uneven maturity of the field: despite nearly two decades of activity, no clear methodological transition between generations has taken place.

Two methodological concerns recur. First, a same-substrate expert reference is used in three studies [48,55,76], where the AI is judged against a human reading of the same substrate it processes (human KOH versus AI on KOH, or clinician classification of fluorescence versus AI on fluorescence); this is not, in itself, circular, but it caps the demonstrable performance, since the model can at best reproduce a fallible reading of that substrate. Second, the Rajitha cluster [77,78,79], three successive publications by one group, shows partial or complete dataset overlap without explicit disclosure, and one study [78] states that the code is unavailable.

Three D2 studies stand out as comparatively more mature in methodological terms and are appraised for clinical readiness in Section 5.5.1. Decroos et al. [49] is the only D2 study with formal non-inferiority testing (Bonferroni correction, reported 95% CIs, a four-dermatopathologist comparator on N = 199) and avoids the same-substrate limitation through a composite standard (pathologist consensus plus dermatophyte PCR for the 49 uncertain cases). He et al. [74] uses a composite clinical, mycological, and dermatological reference, with the evaluated tool excluded from it to avoid incorporation bias, and comparators quantified on the same N = 300. Hulse et al. [75] is the corpus’s only preregistered study (OSF), with consecutive selection by exposure (clinically ordered PAS testing rather than by final diagnosis), an independent PAS reference and Firth-penalised confidence intervals suited to rare events (N = 8 PAS-positive); it is, strictly, a feature-based predictive model rather than an image-analysis tool, in which human assessors score the histopathologic clues and the machine-learning step maps a single clue (possible hyphae) onto the probability of PAS positivity. Three further studies are notable for other reasons: Berger [54] reports the only technical failure rate above 10% in the corpus (30% of nail clippings unusable); Köberle [22] is the only D2 study dedicated to *Malassezia*, with a pure ViT trained on 1113 microscopic fields; and Gagna et al. [72] has the smallest sample in the corpus (14 patients), which limits its generalisability.

#### 5.3.3. D3—Analytical, Molecular

At the input level, D3 is the most heterogeneous domain in the corpus: its four studies share no common data type, spanning DNA microarray, mass spectrometry and sequence-based classification. Despite being the smallest domain, it carries disproportionate clinical weight, because species identification and antifungal-resistance prediction are the analytical steps with the most direct therapeutic consequences, particularly given the global emergence of terbinafine-resistant *Trichophyton indotineae* [8].

D3 also shows the highest concentration of industry ties of any domain, three of its four studies carrying a graded conflict of interest (Table S5). This is a structural concern precisely where the therapeutic stakes are greatest, since a developer-run evaluation of a resistance-typing platform is exactly where optimistic reporting would matter most. Against this background, Tirard-Collet [61] stands out as the domain’s clearest independent evaluation: performed on a French population with no platform-affiliated authors, it surfaced a real-world technical-failure rate for the commercial kit, the kind of reliability figure that developer-run studies rarely report. Ivagnes [62], by contrast, recorded its ethics review as “not applicable” despite using patient specimens, which is questionable under current standards.

Todd [63] has the most complete transparency profile in the corpus (public code, public data, a distinct-patient count of 188 and no conflict of interest). Its wider interest is that the study, though not the model itself, connects identification to treatment: the CNN only assigns each isolate to one of 28 TiTmSC genotypes, susceptibility testing is performed separately and the genotype then maps onto the resistance profile, with *Trichophyton indotineae* carrying terbinafine resistance through SQLE mutations (L393S, F397L) while *T. mentagrophytes* genotypes VII and II* remain susceptible. This data-level chain from genotype to resistance to azole treatment is what makes it a partial, implicit D3-to-D4 interface rather than an integrative tool itself.

#### 5.3.4. D4—Declared Empty: A Structural Finding

The integrative post-analytical step (D4) is where an analytical result becomes a clinical decision: interpretative reporting, antifungal-resistance alerting, and therapeutic steering. This step is not a national particularity but the post-examination phase of the internationally standardised total testing process; ISO 15189:2022 requires the laboratory to describe the interpretative and advisory services it offers to patients and requestors and lists post-examination interpretation among its reporting requirements [84]. The function is performed by a medical biologist in France and much of continental Europe (the biologiste-conseil), by a consultant clinical scientist or medical microbiologist in the United Kingdom and, where no formal laboratory-consultation role exists, largely by the treating clinician; defining D4 by this function rather than by a single national role is what keeps the framework portable. As of corpus closure, as far as we know, no AI application dedicated to this step for superficial mycoses had been published; this emptiness is not an artefact of our eligibility criteria nor an oversight in searching, but a structural finding about the field, and the most consequential one in this review.

The reason the step is empty is also the reason it is hard, and it bears on who integrates the data at all. D4 is the only step that must combine inputs of different kinds, the analytical result, the requesting clinical context, host and epidemiological factors and prior treatment, into a single actionable judgement. In current practice, no single actor holds all of these: the laboratory issues an interpretative comment from what it can see, while the treating clinician makes the therapeutic decision from the clinical picture, so the integration is split between roles and rarely assembled in one place. Only two studies touch D4 at all, and both incidentally, Hulse [75] reaching from D2 toward a downstream laboratory decision and Todd [63] from D3 toward treatment selection (Section 5.1 and Section 5.3.3). The imaging-centred studies that dominate the corpus operate on one homogeneous data type and never confront this integration. An AI addressing D4 would require structured, linked clinical and laboratory data that current datasets do not contain, which is why the gap is unlikely to close simply by scaling the prevailing image-classification paradigm. A future D4 application would therefore look less like the clinician-facing image tools that dominate the corpus and more like an orchestration layer over laboratory output: on receipt of a species identification and, where available, a susceptibility result, it would assemble a structured interpretive report for the prescribing clinician. The nearest existing footholds are not new architectures but modest extensions of tools already in the corpus, for instance, turning a histopathology decision-support output into a structured report that flags when an additional stain or an atypical entity warrants attention. We frame this as a direction rather than a demonstrated possibility; nothing in the corpus yet establishes that such integration performs adequately.

### 5.4. Cross-Cutting Methodological Limitations

Critical reviews of AI in superficial mycoses converge on a familiar set of weaknesses: small single-centre datasets, limited external and prospective validation, binary framings and sparse calibration and explainability reporting [23,36,39]. Rather than restate these, we quantify them across the whole D1–D4 partition, not onychomycosis alone, and add the domain-specific weaknesses a nail-focused literature could not surface.

#### 5.4.1. Internal Validity: Data Leakage and Outcome-Reporting Bias

The most serious internal-validity threat here is not small samples but undeclared data reuse. When successive publications from one group share data while presenting a “new” evaluation, apparent independent replication is illusory and measured accuracy is optimistic by construction, a recognised driver of the reproducibility crisis in which patient-level information leaks across training and test partitions and inflated results propagate because non-replicable work tends to be cited more, not less [83]. The corpus contains such clusters, none declaring the overlap in their Methods (Table 4). Compounding this, prospective preregistration, the standard safeguard against outcome-reporting bias, is essentially absent (a single study), leaving the remainder unassessable on that axis. Neither problem shows up in a summary metric such as AUC; detecting either requires close reading of each study’s methods. As a scoping review, ours can flag and quantify these issues but cannot substitute for the study-level appraisal they require.

#### 5.4.2. Findings on External Validity and Reproducibility

Even where internal estimates are sound, they rarely travel; complete external validation is the exception, the highest headline figures rest on internal validation, and the two algorithms re-evaluated on new populations both degraded on transfer, the expected direction when a model leaves the data it was tuned on. This is the local form of a pattern documented across medical-imaging AI, where high internal accuracy repeatedly fails to reach independent clinical use [3], and which AI-specific extensions such as QUADAS-AI were designed to surface [33]. Transfer is further limited by whom the datasets represent: demographic characteristics are unreported in close to half the corpus, and no D1 study stratifies performance by Fitzpatrick phototype, so performance across skin tones is unknown for these tools rather than shown to be worse, an omission that is not benign given evidence that dermatology AI can perform unevenly across skin tones [82]. A reported AUC therefore describes a dataset more than a test. Reproducibility is scarcely better: only a handful of studies release both code and data, and real-world feasibility is hard to judge when the technical-failure rate is almost never reported.

#### 5.4.3. Conflicts of Interest

We did not apply a validated conflict-of-interest instrument, because we are not aware that such an instrument exists; what we report is a transparent, descriptive account of declared affiliations and their relation to the specific tool or reagent each study evaluates (Table S5). Two observations follow. First, industry ties are not evenly spread but concentrate in the molecular domain (D3), where the evaluated products are commercial identification and resistance-typing platforms, the configuration in which favourable results are most consequential and independent verification scarcest. The distortion is not confined to any single paper: industry-supported research accrues disproportionate citations and becomes financially entangled with the journals that carry it [85], so that, together with the tendency of non-replicable findings to be cited more rather than less [83], commercial involvement can inflate a technology’s apparent standing well beyond its evidence. Second, and less often noted, a conflict of interest can raise measured accuracy through a mechanism unrelated to reporting: when the people preparing specimens, operating the instrument and interpreting its output are the tool’s own developers, the study captures the system at its expert-user ceiling. The error rate suppressed by that familiarity is precisely what returns when the tool is handled by ordinary clinicians or technicians, so developer-run evaluations measure a best case that routine practice cannot reproduce, an optimism that is intrinsic and non-transferable, and that bites hardest in D3, where specimen preparation and signal interpretation are most demanding. This is why the corpus’s few genuinely independent evaluations carry weight out of proportion to their number: they are the ones that report the unglamorous figures, technical-failure rates and degraded external accuracy, that developer-run studies rarely surface. The aim is not to impugn individual studies, several of which declare their affiliations fully, but to note that the domain most entangled with industry is also the one where the evidence most needs, and least often receives, outside confirmation.

### 5.5. Implications for Current Clinical Practice

A scoping review maps the literature and its gaps; it does not grade certainty of evidence or certify tools for deployment, and we therefore stop short of designating any application as ready for clinical use. What the map does support is a more modest statement: where, and for which task, the reported evidence is currently strongest, and what would have to be shown before use.

#### 5.5.1. Where the Reported Evidence Is Strongest

In our opinion, the most rigorously evaluated application in the corpus is Decroos et al. [49], which tested non-inferiority against several dermatopathologists with confidence intervals and a composite reference standard for uncertain cases; on current evidence, it appears the most credible candidate for evaluation as a second reader in routine PAS histopathology, though its validation remains single-centre and internal, so independent external testing would be a precondition for use. He et al. [74], which used a composite clinical-mycological reference with the fluorescence method excluded to avoid incorporation bias, may be a plausible adjunct to manual KOH and fluorescence microscopy in high-volume laboratories, again pending external validation. Todd et al. [63] is, in our view, better understood not as a bedside diagnostic but as a reference-quality tool for epidemiological surveillance and resistant-genotype identification, of particular value given the emergence of terbinafine-resistant *Trichophyton indotineae*. We do not present any of the three as clinically validated for implementation; each marks, rather, where the evidence has so far advanced furthest.

#### 5.5.2. The D1 Smartphone-Clinician Domain: Triage Rather than Replacement

Reported accuracy in the clinician-facing D1 domain is high, but it rests disproportionately on internal validation, on a small number of proprietary algorithms recurring across studies and on datasets whose phototype composition is largely unknown, with no D1 study stratifying performance by Fitzpatrick type. The empirical case for caution is the degradation seen when these algorithms are re-evaluated on new populations. Taken together, this makes it unlikely that a commercially available smartphone application can replace clinical examination followed by mycological confirmation. Their defensible role is narrower, triage, flagging whether a lesion warrants paraclinical confirmation, used with explicit awareness that their performance across skin phototypes remains largely uncharacterised.

#### 5.5.3. Post-Publication Independent Evaluation as a Methodological Model

The most encouraging methodological signal in the corpus is a small set of studies that re-evaluated a previously published algorithm independently, by a separate team or on a new population and quantified how much performance changed. One such evaluation documented a real-world technical-failure rate several times higher than the original developer report had implied, rather than confirming it. This is categorically different from re-analysis by the same group on overlapping data, and it is the mechanism by which a field moves from isolated high-accuracy reports to cumulative, externally grounded evidence. Encouraging it, through editorial expectations for independent validation or through preregistered validation studies designed as primary aims, is a concrete and low-cost step toward maturity, consistent with the principle that diagnostic AI should ultimately be judged by clinical utility rather than by internal-validation performance alone [1].

### 5.6. Recommendations for Future Research

**Figure 2:**
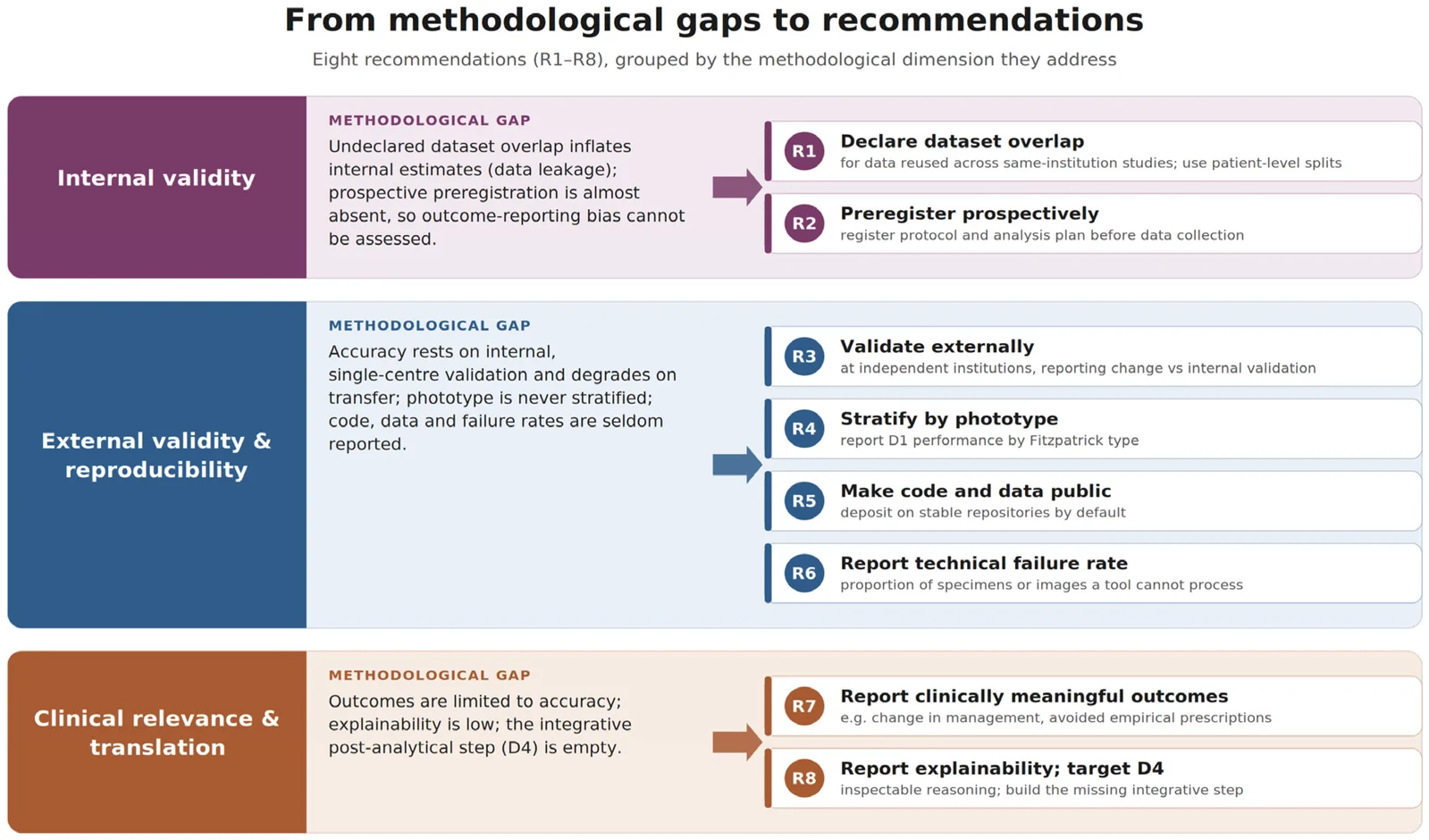
From methodological gaps to recommendations. The eight recommendations (R1–R8) arising from this review, grouped by the methodological dimension of the gap each addresses: internal validity (R1–R2), external validity and reproducibility (R3–R6) and clinical relevance and translation (R7–R8). D1 = clinician-facing image analysis; D4 = integrative post-analytical step.

The gaps identified in this corpus are methodological rather than technological, and they point to eight recommendations grouped by the methodological dimension each addresses (Figure 2). For internal validity, any substantial dataset overlap with a group’s earlier work should be declared, since undeclared reuse inflates apparent performance, and studies should be preregistered prospectively, the standard safeguard against outcome-reporting bias and one met by only a single study here. For external validity and reproducibility, evaluation should move to independent institutions with performance reported against internal validation, performance should be stratified by skin phototype, public code and data should be the default and the technical failure rate should be reported. For clinical relevance and translation, studies should report outcomes with clinical meaning rather than accuracy alone and make their reasoning inspectable while treating the integrative post-analytical step (D4) as an explicit target, since low explainability remains a barrier to adoption [39]. Beyond these study-level actions, the logical next step for the field is a systematic review of specific entities, such as AI for onychomycosis, with meta-analysis where the primary studies permit.

### 5.7. Limitations of This Scoping Review

The principal methodological limitation is the absence of a validated, finalised instrument for appraising AI-based diagnostic-accuracy studies. QUADAS-2, which we used as an organising taxonomy, was developed for conventional diagnostic-accuracy research and does not capture criteria specific to machine-learning work, including the adequacy and reporting of training data, strict separation of training, validation and test partitions, the risk of overfitting on small datasets, external validation and bias arising from homogeneous training populations. The QUADAS-AI extension was designed to close exactly these gaps [33,35] but was not available in finalised, consensus form while this review was conducted. The lack of a unified appraisal standard for AI diagnostic-accuracy studies in mycology is itself a gap in the field and a target for dedicated methodological work.

The corpus and the search carry their own limitations. Gray literature, preprints and minor conference proceedings were not searched systematically, consistent with scoping-review practice in fields whose primary studies appear largely in indexed journals; Scopus and Web of Science were not queried, and forward citation chasing on the included studies was not performed, so a small number of eligible studies may have been missed. The restriction to English-language publications may have excluded studies available only in other languages, notably Chinese and Korean, a non-trivial risk given the corpus’s concentration in east and south Asia. The closing date is a fixed point in a fast-moving field, and work published afterwards is necessarily absent. Finally, the review protocol was not preregistered, and screening and selection were carried out by a single author, both of which limit the reproducibility of the selection process.

The D1–D4 partition is a single-axis simplification—it makes the landscape legible but flattens the nuance of studies that sit between steps. Alternative allocations of the borderline cases are defensible; each allocation decision is documented so that readers can judge it independently.

## 6. Conclusions

AI for the diagnosis of superficial mycoses is expanding rapidly but unevenly across the steps of the diagnostic pathway. Reported accuracy is often high but rests mainly on internal, single-centre validation with limited reproducibility, and two under-recognised threats further weaken the evidence base: undeclared dataset overlap between successive same-institution publications, and a concentration of industry-linked conflicts of interest in the molecular domain, precisely where independent verification is scarcest. External validation at independent institutions, reproducibility through open code and data and the development of the missing integrative step are the priorities for useful clinical translation.

## Figures and Tables

**Table 2:** The four domains of the D1–D4 operational grid (summary).

Domain	Input	Intended User	Output
D1—Clinical, pre-diagnostic step	Clinical image (photograph, dermoscopy, vision-language model)	Clinician (dermatologist, family physician)	Diagnostic prediction, triage support
D2a—Direct microscopy	Fresh preparation (KOH, calcofluor, tape strip)	Microbiologist, mycologist	Fungal-element detection
D2b—Histopathology	Fixed tissue (PAS, GMS, H&E, Grocott, whole-slide imaging)	Pathologist, dermatopathologist	Fungal-element detection on section
D2c—Culture reading	Colony (macroscopic and microscopic morphology)	Microbiologist, mycologist	Visual colony identification
D3—Molecular analytical step	Molecular signal (MALDI-TOF spectrum, ITS/TEF1 sequence, microarray, e-nose)	Mycological reference laboratory	Species/genotype identification, resistance profile
D4—Integrative post-analytical step	Data ensemble (laboratory result + clinical picture + epidemiological context)	Clinical biologist, prescribing clinician	Interpretive report, resistance alert, therapeutic recommendation

## Data Availability

No new data were created or analysed in this study. Data sharing is not applicable to this article.

## Supplementary Materials

The following supporting information can be downloaded at: https://www.mdpi.com/article/10.3390/jof12090684/s1, Table S1: General characteristics of the included studies; Table S2: Internal validity of the included studies; Table S3: External validity and reproducibility of the included studies; Table S4: Search queries; Table S5: Conflicts of interest.
