# Supplementary material for: Artificial Intelligence in the Diagnosis of Superficial Mycoses: A Scoping Review
Source: J Fungi (Basel). 2026 Sep 11;12(9):684. doi: 10.3390/jof12090684 (PMC13608722; doi:10.3390/jof12090684)
Supplement: Supplementary file 1 [file jof-12-00684-s001.zip › Supplementary_Tables_S1_S3_revised_refs.pdf]

Supplementary Tables S1-S3. AI applications for the diagnosis of superficial mycoses (n = 33 primary studies).

Corrections introduced in this revision are shown in red. "[!]" marks a caution note. Ranges and hyphens use "-"; magnification is written "x".

Supplementary Table S1. General characteristics of the included studies.

D1 - pre-analytical clinician-facing image processing (n = 13); D2 - analytical microscopy/histopathology (n = 16); D3 - analytical molecular techniques (n = 4); D4 - integrative post-analytical (DECLARED EMPTY, n = 0; partial cases: Hulse2026 in D2, Todd2026 in D3).

Domain	Study	Year	Country	Setting	Design	AI-system note
D1	Duzayak et al [56]	2023	Turkey	Secondary hospital - Sakarya Karasu Public Hospital, Dermatology & Venereal Diseases Outpatient Clinic	Prospective consecutive; diagnostic accuracy	Classical ML (ANN, SVM, EDT); clinical toenail images; onychomycosis vs. healthy; 300 features/image (4-channel HSV pipeline + Eta selection); clinical hardware constraints; collection period 02/01/2020 - 02/01/2021.
D1	Han et al [47]	2018	South Korea	Tertiary hospital - Asan Medical Center (dev.); Inje Univ., Hallym Univ., Seoul Natl. Univ. (multi-site external validation)	Retrospective (dev.) + multi-site external validation	CNN ensemble (ResNet-152 + VGG-19 + FF-NN); nail images; onychomycosis vs. nail dystrophy; R-CNN auto-labeling from archive (49,567 img.); public model (figshare + nail.medicalphoto.org).
D1	Idress et al [64]	2025	Sudan	Tertiary hospital - Khartoum Teaching Hospital of Dermatology and Venereology, Khartoum	Prospective cross-sectional	TL-LGBM ensemble (Inception-ResNet-v2 + DenseNet121 + EfficientNetB0 + LightGBM on DWT features); nail images; onychomycosis vs. dystrophy vs. normal; limited-resource context.
D1	Julanon et al [65]	2025	Thailand	Tertiary hospital - Srinagarind Hospital (KKU, Khon Kaen) + Northeast Thailand teledermatology	Retrospective, diagnostic	Multi-task CNN (classification + segmentation, adaptive weighting); clinical images; tinea corporis/cruris vs. non-dermatophytoses; skin of color (Fitzpatrick III-IV); public GitHub code.
D1	Kim et al [66]	2020	South Korea	Tertiary hospital - Asan Medical Center, Univ. of Ulsan (Seoul)	Prospective observational comparative	Three-stage deep CNN (Faster-RCNN/VGG-16 detector + 2x ResNet-152 classifier); clinical nail photographs taken by non-physicians; onychomycosis vs. onychodystrophy; pre-existing Han2018c algorithm.
D1	Kong et al [57]	2025	China	Tertiary hospital - Sun Yat-sen Memorial Hospital, Sun Yat-sen University, Guangzhou	Retrospective, model development/validation	HybirdRVIT-CNN (Vision Transformer + CNN, Adaptive Attention Module); dermoscopic images; onychomycosis vs. other nail disorders vs. healthy; reference standard: expert labeling (no KOH/culture). [!] Dataset overlap with Zhu2022: same institution (Sun Yat-sen Memorial, Guangzhou), same period May 2019 - February 2021, 1,155 images with identical breakdown (603 OM + 221 psoriasis + 104 dystrophy + 227 normal).
D1	Mehta et al [67]	2024	India	Tertiary hospital - AIIMS New Delhi (academic)	Prospective cross-sectional	DermaAId (DenseNet-161, Nurithm Labs); clinical photographs; differential diagnosis of genital dermatoses (40 classes); mycoses = 12.5% subset (tinea cruris n = 15, candidiasis n = 9); algorithm from Pangti2021.
D1	Miura et al [58]	2024	Japan	Tertiary hospital - Foot Care Outpatient Clinic, Akita University Hospital	Retrospective, model development/validation	Four-stage pipeline (YOLOv8 detection + SwinIR super-resolution + U2-Net segmentation + ViT-B classification); standardized nail photographs; tinea unguium vs. normal; telemedicine/diabetic-foot context.
D1	Nigat et al [68]	2023	Ethiopia	Primary care - Dr. Gerbi Medium Clinic, Jimma	Retrospective, CNN model development	Custom CNN (HSFDCM); clinical photographs; 4-class tinea classification (capitis/pedis/corporis/unguium); limited-resource screening; reference standard: dermatological diagnosis (no KOH/culture mentioned).
D1	Pangti et al [69]	2021	India	Mixed: Tertiary hospital - AIIMS New Delhi (N = 3,699); rural primary care - Jhajjar (N = 932); private urban community - Gurugram (N = 383)	Prospective cross-sectional (clinical validation)	Modified DenseNet-161 (DermaAId, Nurithm Labs); smartphone clinical images; 40 dermatological classes; mycoses = subset (tinea cruris/corporis best represented); 5,014 patients - largest N in the corpus.
D1	Police et al [70]	2025	India	Tertiary hospital - Sathya Hospital and Sathya Diagnostics, Hyderabad	Prospective cross-sectional	3 commercial AI apps compared (Aysa/VisualDx, Skinner, AI Dermatologist Skin Scanner); clinical images; 5 classes (tinea corporis = subset, n = 8); the only study using commercial apps.
D1	Yodrabum et al [59]	2026	Thailand	Tertiary hospital - Siriraj Hospital, Mahidol University, Bangkok	Mixed retrospective + prospective (Siriraj dataset + DermNet + new collection)	Benchmark of 16 architectures (8 CNN + 8 Transformer); best: Swin Transformer; smartphone clinical images; dermatophytoses vs. eczema vs. psoriasis; Grad-CAM; patient N NR (heterogeneous mixed dataset).
D1	Zhu et al [71]	2022	China	Tertiary hospital - Sun Yat-sen Memorial Hospital, Guangzhou	Retrospective, model development/validation	Ensemble of 3x Faster R-CNN (TensorFlow); dermoscopic images; onychomycosis vs. nail psoriasis vs. traumatic onychodystrophy; robust reference standard (KOH+ and/or culture+ and/or PAS+); 54 dermatologist comparators. [!] Dataset overlap with Kong2025: see detailed note at Kong2025.
D2	Berger et al [54]	2007	Netherlands	Laboratory (referral)	Interobserver reproducibility study (prospective)	PAPNET/NNS (pre-deep-learning); PAS histopathology (nail clippings); 50 cases; interobserver reproducibility (kappa 0.70-0.97 diagnostic, 0.53-0.79 tile-based; SE 0.1 and 0.08 respectively); Se/Sp/AUC NR. [!] Reproducibility study, not accuracy.
D2	Decroos et al [49]	2021	Germany	Tertiary hospital	Retrospective, diagnostic accuracy (non-inferiority)	CNN (VGG-13-like, patch-based) on PAS WSI (nails); N = 199 patients; Se = 94.1%, Sp = 98.0%, AUC = 0.981; non-inferior to 4 dermatopathologists; composite reference (PAS + selective PCR).
D2	Gagna et al [72]	2024	USA	Laboratory (referral)	Descriptive-methodological (B-DNA IHC + WSI + AI)	HALO AI v3.5 + ImageJ; anti-ds-B-DNA IHC on FFPE; N = 14 (all T. rubrum, ages 6-24 years); AI accuracy 82% on training images (no independent test set); [!] IRB undeclared; commercial specimens (Ideal Labs).
D2	Gao et al [73]	2021	China	Tertiary hospital	Prospective validation study	ResNet-50; UV fluorescence microscopy (340 nm); N = 292 specimens; Se = 96.8%, Sp = 93.2%; reference standard = human microscopy (not culture); Wangsheng stain (proprietary). [!] IRB undeclared.
D2	He et al [74]	2025	China	Tertiary hospital - Shanghai Skin Disease Hospital, Department of Mycology	Prospective diagnostic-accuracy study	FMIA (Model FA500, Chongqing Tianhai) - automated CNN fluorescence-microscopy system; chitin-specific fluorescent stain; N = 300 patients; Se = 96.27%, Sp = 94.92%, AUC = 0.96; comparators: manual KOH (Se = 75.52%, AUC = 0.84) and manual fluorescence (Se = 92.95%, AUC = 0.95).
D2	Hulse et al [75]	2026	USA	Tertiary hospital - Univ. of Missouri-Kansas City, School of Medicine (academic dermatopathology)	Retrospective cohort (consecutive selection by exposure - PAS testing)	ML (Firth logistic regression, RF, SVM, KNN); H&E histopathological features; single significant predictor (possible hyphae); P(PAS+ | hyphae) = 0.93.
D2	Jansen et al [50]	2022	Germany	Laboratory (referral), multicenter - 4 dermatohistopathology laboratories (Germany + Austria)	Retrospective, multicenter, diagnostic accuracy	U-NET + ResNet50; PAS WSI (nails); 4 laboratories, 3 scanners; N = NR (664 WSI); Se = 94%, Sp = 77%; reference = consensus of 11 dermatopathologists from 10 institutions; AI accuracy 86.49% vs. pathologist median 87.84%. [!] CoI: Aignostics GmbH (co-author).
D2	Köberle et al [22]	2024	Germany	Tertiary hospital	Classifier development and validation study	Vision Transformer ViT-B/32 (best); native tape strip (brightfield, no KOH); Malassezia spp. / PV; N = NR (22 specimens, 1113 FoV); Se = 94.0%, Sp = 93.5%, AUC = 0.99; image-wise split -> data-leakage risk.
D2	Koo et al [76]	2021	South Korea	Secondary hospital - Veterans Health Service Medical Center, Seoul	Retrospective, object-detection model development	YOLO v4; KOH microscopy (10%, 40x and 100x); N = 38 patients (18 positive: 10 at 40x + 8 at 100x; 20 negative at each magnification); Se = 93.2-99.0%, Sp = 86.6-100%, AUC = 0.965-0.999 (per dataset); [!] small N; circular reference (human KOH = the test evaluated by the AI).
D2	Mäder et al [55]	2015	Germany	Tertiary hospital (university)	Algorithm development and validation study	Traditional image processing (Canny edge, mathematical morphology, rule-based classification; no ML/DL); fluorescence (MykoColor); N = NR (415 images); Se = 83%, Sp = 79%. [!] Oldest D2 study; non-transferable thresholds; IRB NR.
D2	Rajitha et al [77]	2022	India	Tertiary hospital - Kasturba Medical College, Manipal (DVL Clinic)	Retrospective, CNN development (conference paper)	Transfer learning: EfficientNetB0 (best), VGG16, ResNet50, MobileNet; KOH bright-field; N = NR (2626 patches); EfficientNetB0: Se = 97.64%, Sp = 99.40%, AUC = 0.99. [!] Dataset overlap with Rajitha2025/2025a.
D2	Rajitha et al [78]	2025	India	Tertiary hospital - Kasturba Medical College, Manipal (DVL Clinic)	Semantic-segmentation study (methodological)	U-Net with residual modules, SE, and Attention Gating; KOH bright-field (10x and 40x); N = NR (patches); Dice = 76.41 (10x) / 84.61 (40x); Recall = 75-78%, Sp = 96-97%. [!] Pixel-level metrics, not per-patient; dataset overlap with Rajitha2022/2025a.
D2	Rajitha et al [79]	2025	India	Tertiary hospital - Kasturba Medical College, Manipal (DVL Clinic)	Quantitative grading study (methodological)	U-Net + C-ROC statistical model; fungal-load grading (low/moderate/high); KOH bright-field; N = NR (378 images); Se = 80-100%, Sp = 92-96% per grade; Fleiss kappa 0.847 (automated vs. manual).

Domain	Study	Year	Country	Setting	Design	AI-system note
D2	Rappoport et al [80]	2024	Israel	Tertiary hospital - Sheba Medical Center, Ramat Gan	Retrospective (archival biopsies 2014-2023)	DeePathology STUDIO (CNN, Tiles Mode); WSI of PAS/GMS-stained skin biopsies; detection of microscopic fungal elements; unit of analysis = tiles; patient N NR (22 slides: 7 PAS + 15 GMS); POC; CoI: 3 authors DeePathology Ltd.
D2	Ren et al [81]	2025	China	Tertiary hospital - Huashan Hospital, Fudan University, Shanghai	Dual-model AI development and evaluation	YOLOX-L (hyphae/spore detection) + MobileNet V2 (mycelium); fluorescence (Jiangsu Life Time stain); N = NR (942 images); Recall = 99.3%, Precision = 92.5%, Kappa = 0.857. [!] CoI: 3 co-authors SODA Data Technology + 2 co-authors 3D Medicines.
D2	Yilmaz et al [48]	2022	Turkey	Secondary hospital - Haydarpaşa Numune Training and Research Hospital, Istanbul	Case-control accuracy + human comparison (16 dermatologists)	VGG16 and InceptionV3; grayscale KOH (no stain, 40x); N = NR (10 source patients; metrics on 1415 patches); VGG16: Se = 74.93%, Sp = 93.78%, AUC = 0.934; comparator: 16 dermatologists (mean accuracy 74.53%). [!] Circular reference.
D3	Anton et al [60]	2023	France	Laboratory (referral) - INOVIE network (Toulouse, Montpellier)	Prospective-retrospective technology validation study	DNA microarray (DendrisCHIP®DP) + HOMER + multilabel Random Forest (DendriSOFT®); 284 clinical specimens; Se = 83-100% per species, Sp = 99-100%; internal validation only; [!] Maximal CoI: authors = employees/founders of Dendris SAS.
D3	Ivagnes et al [62]	2025	Italy	Tertiary hospital, multicenter - Rome + Vicenza (Italy) + Delhi (India)	Retrospective, MALDI-TOF ML validation study	PLS-DA, SVM, RF, LightGBM, KNN on MALDI-TOF spectra (Clover MSDAS v1.10.0); N = 56 isolates (3 centers); balanced accuracy = 100% (PLS-DA/SVM, 10-fold CV); biomarker AUC at 3423.53 m/z = 1.0. [!] CoI: co-author Arroyo = Clover Bioanalytical Software; IRB declared "Not applicable."
D3	Tirard-Collet et al [61]	2025	France	Tertiary hospital - Hospices Civils de Lyon (Hôpital Croix Rousse)	Retrospective validation study	DendrisKIT®DP: pan-fungal PCR + microarray + HOMER + multilabel RF (DendriSOFT® v1.0.0); N = NR (85 specimens); Se = 83.9% (95% CI: 72.8-91.0), Sp = 88.9% (95% CI: 67.2-96.9); technical failure rate 5.9% (5/85 invalid PCR). Independent evaluation of Anton2023. [!] Co-author C. Miossec = author of the 2026 anchor reference.
D3	Todd et al [63]	2026	USA	Laboratory (referral) - Wadsworth Center Mycology Laboratory, NYS DOH, Albany	Retrospective epidemiological surveillance + CNN development/validation	Deep-learning CNN (6-mer counts, ITS Sanger, 28 TiTmSC genotypes); N = 188 distinct patients; concordance 98.7% (382 isolates); Se/Sp NR (% concordance); CDC funding; no declared conflict. [!] Implicit D3->D4 interface via genotype -> AFST resistance -> therapeutic-decision correlation.

Supplementary Table S2. Internal validity of the included studies.

N = distinct (real, human) patients; studies reporting only images/specimens/patches/isolates/slides have N = NR. Primary metric: AUC preferred; if AUC absent, Accuracy or Balanced Accuracy. Se = sensitivity; Sp = specificity.

Domain	Study	Reference standard	Patient N	Validation type	Human comp.	Primary metric / Comments	95% CI	Declared threshold / Comments
D1	Duzayak et al [56]	Specialist clinical diagnosis (no KOH/culture)	76	Internal	No	AUC 0.97 (best: EDT L18); Se 0.96; Sp 0.98; Acc 97.25%. Comment: split by images, not by patients.	NR	No (implicit threshold); value undeclared.
D1	Han et al [47]	KOH/culture/antifungal response (per dataset B1-D)	4557 (dev.) + 342 (external validation)	Multi-site external (3 independent institutions)	Yes	AUC 0.98 (B1) -> 0.82 (D); Se 96.0% -> 87.7%; Sp 94.7% -> 69.3%. Comment: degradation on external validation; ensemble ResNet-152 + VGG-19 + FF-NN.	Yes (mean ± SD)	Yes - Youden index (max Se+Sp); explicitly pre-specified threshold.
D1	Idress et al [64]	KOH + fungal culture (clinical and lab. confirmation)	350	Internal	Yes	AUC 0.955; Se 95.45%; Sp 91.59%; Acc 96.8%. Comment: train/test split NR.	NR	No; threshold undeclared.
D1	Julanon et al [65]	KOH / histo / culture (derm.); consensus >=2/3 derm. (non-derm.)	580	Temporal external	Yes	AUC 0.84 (95% CI 0.80-0.88); Se 0.80; Sp 0.71; F1 0.71; NPV 0.83. Comment: temporal validation, not institutional.	Yes	Yes - threshold 0.5 (implicit); AUC computed on full ROC.
D1	Kim et al [66]	KOH 40% + fungal culture (positive if at least one +)	90	Independent prospective (external algorithm)	Yes	AUC 0.751 (95% CI 0.646-0.856); Se 70.2%; Sp 72.7%. Comment: pre-existing Han2018c algorithm.	Yes	External - derived from Han2018c (max Se+Sp).
D1	Kong et al [57]	Expert labeling (no KOH/culture - major limitation)	NR (1155 images)	Internal	No	AUC 0.877 (OM vs. other disorders); AUC 0.939 (disease vs. healthy). Comment: patient N NR; weak reference standard.	NR	No (implicit); value undeclared.
D1	Mehta et al [67]	Consensus >=2 board-certified derm. + laboratory or histo	257 (of 834 screened)	Independent prospective (external algorithm)	Yes	AUC 0.99 (tinea cruris); AUC 0.68 (candidiasis); Se tinea cruris top-1 93.3%; Se candidiasis top-1 33.3%. Comment: mycoses = 12.5% subset; Pangti2021 alg.; 4 comparators (2 PCP + 2 derm).	NR	External - cut-off 40% (derived from Pangti2021).
D1	Miura et al [58]	Medical clinical evaluation (expert labeling; no KOH/culture)	50	Internal	No	Recall 0.907; F1 0.898; Acc 0.897; AUC NR. Comment: N = 50; massive augmentation (551 -> ~20000); weak reference standard.	NR	Implicit 0.5; authors suggest 0.3.
D1	Nigat et al [68]	Dermatological diagnosis (confirmation method NR)	NR (407 images)	Internal	No	Se 86.4%; Sp 95.4% (overall, 4 classes); val. accuracy 87.3%; AUC NR. Comment: patient N NR; weak reference standard; split NR; data-leakage risk from augmentation before split.	NR	No (softmax 0.5 implicit).
D1	Pangti et al [69]	Consensus >=2 board-certified derm. (no systematic KOH/culture)	5014 (3699 tertiary + 932 rural + 383 private urban)	Internal	No	AUC 0.95 (tinea cruris/corporis); AUC 0.75 (tinea pedis); AUC 0.81 (candidiasis); overall top-1 75.07% (95% CI 73.75-76.36); Se tinea pedis 40.0%; Se candidiasis 41.18%. Comment: largest N; superior performance in private urban (80.3%) vs. tertiary (74.9%) top-1.	Yes	Partial - variable ROC threshold; explicit fixed cut-off NR.
D1	Police et al [70]	Board-certified derm. consensus (no KOH/culture; clinical)	32	Commercial (pre-existing apps)	No	Top-1 tinea corporis: 50-75% (per app); Cohen kappa: 0.28-0.43; AUC NR. Comment: N = 32; purposive sampling; tinea corporis n = 8.	NR	External (implicit, commercial apps); value NR; app versions NR.
D1	Yodrabum et al [59]	Mixed: KOH (Siriraj) + derm. review (DermNet)	NR (mixed dataset)	Internal CV	Yes	Recall 0.980 (derm.); Acc 0.967; AUC NR. Comment: patient N NR; heterogeneous mixed dataset.	NR	No (3-class softmax); numeric cut-off NR.
D1	Zhu et al [71]	KOH and/or culture and/or PAS+ (explicit criteria - robust)	206	Internal	Yes	Youden 0.715 vs. derm. 0.427-0.444 ($p < 0.05$); Se 93.0%; Sp 78.5%; Acc 87.5%. Comment: AUC NR; 54 dermatologist comparators (most robust in D1).	NR	No (implicit, Faster R-CNN); value NR.
D2	Berger et al [54]	Conventional light microscopy (not culture)	NR (50 nail clippings)	Internal (reproducibility)	Yes	Diagnostic kappa: 0.70-0.97 (SE 0.1); tile-based kappa: 0.53-0.79 (SE 0.08); Se/Sp/AUC NR. Comment: reproducibility study, not accuracy; assessors = medical students.	NR	Not applicable - reproducibility study.
D2	Decroos et al [49]	Consensus of 2 dermatopathologists (PAS) + dermatophyte PCR (49 uncertain cases)	199	Internal	Yes	AUC 0.981 (continuous prob.); Se 94.1%; Sp 98.0%; dichotomized AUC 0.960. Comment: AI non-inferior to 4 dermatopathologists.	Yes	Yes - threshold 91%; optimized on training-validation set.
D2	Gagna et al [72]	Conventional histopathology (PAS, GMS, H&E)	14	Internal	No	Accuracy 82% (training images); +21.6% detection vs. GMS (per WSI); Se/Sp NR. Comment: accuracy on training, not an independent test set.	NR	No (implicit OD values); descriptive, not statistical, thresholds.
D2	Gao et al [73]	Human microscopy (2 expert inspectors; not culture)	292	Internal	Yes	Se 96.8% (overall); Sp 93.2%; skin: Se 99.5% / Sp 91.4%; hair: Se 60% / Sp 100%. Comment: reference = human microscopy.	NR	No (implicit softmax); value undeclared.
D2	He et al [74]	Composite standard: clinical + mycological (KOH + fluorescence) + 2 senior dermatologists (FMIA excluded)	300 (241 confirmed SFI)	Internal	Yes	FMIA: Se 96.27%; Sp 94.92%; AUC 0.96. Manual KOH comparator: Se 75.52%; Sp 93.22%; AUC 0.84. Manual fluorescence comparator: Se 92.95%; Sp 96.61%; AUC 0.95. Comment: rigorous composite standard; FMIA excluded from reference.	Partial (Se only [!])	No (3 consecutive positive frames = positive; 36 negative frames = negative); preset parameters; fixed manufacturer cutoff.
D2	Hulse et al [75]	PAS histochemistry (positive/negative for fungal hyphae)	64 (97 biopsies; 8 PAS+)	Internal CV (5-fold)	Yes (4 assessors: student -> resident -> pathologist -> dermatopathologist)	Best model - Firth logistic regression (single predictor): Bal. Acc 0.88 (95% CI 0.70-1.00); Se 0.75; Sp 1.00; F1 0.86; MCC 0.86. Comment: small N (8 PAS+); very wide CIs; self-declared pilot study; partial D4 candidate (decisional output).	Yes (bootstrap 2000 iter.)	Yes - explicit model: $\log(p/1\text{-}p) = -3.58 + 6.14X$; $P(PAS+ \mid hyphae) = 0.93$.
D2	Jansen et al [50]	Majority consensus of 11 certified dermatopathologists from 10 institutions (74 WSI)	NR (664 WSI total; 74 WSI final validation)	Internal	Yes (11 dermatopathologists)	Se 94%; Sp 77%; AUC NR (ROC graphic). AI accuracy 86.49% vs. pathologist median 87.84%. Comment: low specificity; heterogeneity of PAS protocols.	NR	Yes - area threshold >0.25% of tissue; derived on a separate set (78 WSI).
D2	Köberle et al [22]	Manual annotation by expert mycologist (culture not relevant for PV)	NR (22 specimens; 1113 FoV)	Internal	No	ViT-B/32: Se 94.0%; Sp 93.5%; AUC 0.99. Xception: Se 90.2% / Sp 89.8%. EfficientNetB7: Se 86.3%. Comment: image-wise split (not specimen-wise) - data-leakage risk.	NR	No (sigmoid; checkpoint at max val. accuracy); cutoff undeclared.
D2	Koo et al [76]	Human KOH microscopy (2 dermatologists; CIRCULAR reference)	38	Internal	No	Dataset-40: Se 99.0%; Sp 86.6%; AUC 0.999. Dataset-100: Se 95.2%; Sp 100%; AUC 0.997. Comment: small N; circular reference.	NR	Yes - IOU = 0.25; prob. threshold = 0.244; derived via MATLAB perfcurve on the test set.
D2	Mäder et al [55]	Classification by experienced clinicians (not culture)	NR (415 images)	Internal	Yes	Se 83%; Sp 79% (per image); single hyphae: Se 94%; clustered: Se 89%; AUC NR. Comment: thresholds valid only for the optical system used; circular reference.	NR	No (manual thresholds); not transferable to other optical systems.

Domain	Study	Reference standard	Patient N	Validation type	Human comp.	Primary metric / Comments	95% CI	Declared threshold / Comments
D2	Rajitha et al [77]	NR (initial clinical classification; not explicitly stated)	NR (2626 patches)	Internal CV (4-fold)	No	EfficientNetB0: Se 97.64%; Sp 99.40%; AUC 0.99. VGG16: Se 97.35%; Sp 98.51%; AUC 0.98. Comment: patch-level metrics.	NR	No (sigmoid 0.5 implicit); reference standard undeclared.
D2	Rajitha et al [78]	Manual segmentation masks (pixel-wise ground truth)	NR (patches)	Internal CV (k = 5)	No	Dice 76.41 (10x) / 84.61 (40x); Recall 75-78%; Sp 96-97% (pixel-level); AUC NR. Comment: pixel-level metrics, not per-patient.	NR	No (sigmoid 0.5 implicit); early-stopping patience = 7.
D2	Rajitha et al [79]	Manual grading by 2 expert microbiologists (locally validated guidelines)	NR (378 images)	Internal CV (5 C-ROC sets)	Yes	Fleiss kappa automated vs. manual = 0.847 (95% CI 0.761-0.934). LOW: Se 92-100%, Sp 94-96%; HIGH: Se 88%, Sp 93%; C-ROC AUC > 0.88; percentage agreement 85.3%.	Yes	Yes - C-ROC thresholds derived on 80% of the total.
D2	Rappoport et al [80]	Consensus of 2 specialist pathologists (histopathological annotation)	NR (22 slides: 7 PAS + 15 GMS)	Internal	No	GMS: Se 0.93; Sp 0.99; F1 0.95. PAS: Se 0.80; Sp 0.97; F1 0.78. Comment: unit = tiles (2,690 PAS / 75,961 GMS); patient N NR; POC.	NR	No (implicit, DeePathology STUDIO platform); value NR.
D2	Ren et al [81]	Annotation by 2 clinicians (double-checked; inter-rater F1 80-90%)	NR (942 images)	Internal	Yes	Dual framework: Recall 99.3% (148/149 positive); Precision 92.5%; Kappa 0.857; F1 95.7%; AUC NR. YOLOX mAP: hyphae 0.92, spores 0.89. Comment: maximalist threshold; co-author CoI.	NR	No (IOU = 0.1; any positive patch = positive); maximalist cumulative cutoff.
D2	Yilmaz et al [48]	Human KOH microscopy (2 dermatologists >=20 years; CIRCULAR reference)	10 (5 OM + 5 control; 7073 patches)	Internal CV (5-fold)	Yes	VGG16: Se 74.93%; Sp 93.78%; AUC 0.934. InceptionV3: Se 75.04%; Sp 92.67%; AUC 0.929. 16 dermatologists: mean accuracy 74.53 ± 8.57%.	Yes (comparator SD)	No (sigmoid 0.5 implicit); 16 derm. tested on patches (artificial disadvantage).
D3	Anton et al [60]	Mycological culture (COFRAC) + RT-PCR/NGS (discordant)	NR (284 specimens; 87 patients in the swab group)	Internal	No	Se 83-100% per species; Sp 99-100%; overall accuracy 98%; AUC NR. Comment: internal validation only, by the manufacturer; major CoI. The 98% and per-species Se/Sp are LOOCV on the 441-sample training database; concordance with culture on the 284 clinical isolates was only 75% (scraping) to 86% (swab).	NR	No (proprietary DendriSOFT®); cutoff not publicly declared.
D3	Ivagnes et al [62]	ITS sequencing (>=98% BLAST) + ERG1 sequencing	NR (56 clinical isolates; 3 centers)	Internal CV (10-fold)	No	Balanced accuracy = 100% (PLS-DA/SVM); biomarker AUC at 3423.53 m/z = 1.0; algorithm range 88.74-100%. Comment: internal validation only; overestimation risk.	NR	No (threshold 0.5); exception PLS-DA: 3 components.
D3	Tirard-Collet et al [61]	Mycological culture (Sabouraud, 3 weeks) + pan-fungal PCR + Sanger	NR (85 specimens)	Internal (institutionally independent evaluation)	No	Se 83.9% (95% CI 72.8-91.0); Sp 88.9% (95% CI 67.2-96.9); AUC NR. Comment: manufacturer-independent evaluation (Dendris with no participation in design/interpretation); technical failure rate 5.9% (5/85 invalid PCR).	Yes	No (pretrained algorithm; cutoff not described); proprietary.
D3	Todd et al [63]	Manual ITS alignment (CLC Genomics v25) + BLAST (NCBI + Westerdijk)	188 (135 Ti + 39 TmVII + 14 TmII*)	Internal	Yes	CNN vs. manual concordance: 98.7% (382 isolates); Se/Sp NR (reported as % concordance). Comment: the only D3 study with a confirmed distinct-patient N; public CDC funding.	NR	Yes - SoftMax confidence 0.982-1.0; out-of-distribution via Mahalanobis distance (threshold = 1).

Supplementary Table S3. External validity and reproducibility of the included studies.

Demographic data - Yes (age + sex + complete clinical characteristics) / Partial (at least one key variable NR) / NR. Centers - Multi (independent institutions) / Multi-source (same reference center) / Single. Conflict-of-interest relationships are reported descriptively (no validated instrument).

Domain	Study	Patient selection	Demographic data	Centers	Protocol registration	External validation	Public code	Public data	Incl./excl. criteria + Comments (CoI, funding, IRB)
D1	Duzayak et al [56]	Prospective consecutive	Yes	Single	No	No	NR	NR	Age >=18 years; referred to Sakarya Karasu Public Hospital Jan 2020-Jan 2021. Comment: formal exclusion criteria NR; ethics approval Sakarya Univ. (E.13973, 06/11/2019).
D1	Han et al [47]	Retrospective (dev.) + Consecutive (validation)	Yes	Multi	No	Yes - 3 independent institutions (Inje, Hallym, Seoul Natl.)	Yes	Yes	OM: KOH+ or culture+ or response to antifungals. Dystrophy: KOH- or culture- or non-response or response to IL triamcinolone. Code + data: figshare; public model: nail.medicalphoto.org. IRB Asan Medical Center (S2016-2209-0001; 2017-0087).
D1	Idress et al [64]	Prospective consecutive	Partial	Single	No	No	NR	NR	OM: clinical + laboratory confirmation (KOH + fungal culture); structured questionnaire administered. Comment: exclusion criteria not explicitly reported; individual age NR.
D1	Julanon et al [65]	Retrospective	Partial	Multi-source (same reference center)	No	Partial (temporal)	Yes	No	Included: confirmed tinea corporis/cruris. Excluded: tinea faciei (ethical reasons), tinea manus/pedis, images with >=2 Dx in the same frame, poor resolution, patient-identifiable features. Public code: GitHub praisan/Multitask-Dermatophytosis-Diagnosis. Data: on author request. IRB HE654011, Khon Kaen Univ., 26 Apr 2022.
D1	Kim et al [66]	Prospective consecutive	Yes	Single	No	Partial (external algorithm, single-center)	NR	NR	Consecutive patients with toenail dystrophy; externally derived cut-off (Han2018c, 342 patients, max Se+Sp). Comment: 81.8% of patients with onychodystrophy received the wrong antifungal at the first visit. IRB Asan Medical Center no. 2018-1368; written consent.
D1	Kong et al [57]	Retrospective	NR	Single	No	No	NR	NR	Image quality control before inclusion (224x224 px, RGB). Formal exclusion criteria NR. Comment: patient N NR; the study reports only image count (1155 total). Hospital ethics committee approval; anonymized data. [!] Dataset overlap with Zhu2022.
D1	Mehta et al [67]	Prospective consecutive	Partial	Single	No	Partial (external algorithm, single-center)	NR	NR	Included: consecutive patients with genital dermatoses (834 screened; 257 included after exclusions: investigation refusal n = 239, photography refusal n = 124, negative investigations n = 214). Photographs taken by the attending dermatologist (mobile phone min. 10 MP, indoor lighting). Comment: mycoses = 12.5% subset. AIIMS New Delhi Ethics Committee.
D1	Miura et al [58]	Retrospective	NR	Single	No	No	NR	NR	Standardized photographs (illuminance 500-600 lx, distance 15-20 cm); 551 raw images augmented to ~20,000 for training. Comment: exclusively diabetic population - not representative of mycoses in general; internal split NR; mycological confirmation absent. IRB Akita Univ. School of Medicine (Approval #2925, 27 Dec 2022).
D1	Nigat et al [68]	Retrospective	NR	Single	No	No	NR	NR	407 images (120 capitis, 96 pedis, 71 corporis, 120 unguium); augmentation 407 -> 1069; split not explicitly reported. Comment: patient N NR; diagnosis-confirmation method NR; data-leakage risk (augmentation before split). IRB Jimma Univ. Institute of Technology Research Ethics Approval Board.
D1	Pangti et al [69]	Prospective consecutive	Partial	Multi-source (tertiary + rural + private urban)	No	No	NR	NR	5014 consecutive patients with diverse dermatological conditions (40 classes); single image/patient, photographed by a physician with a smartphone. Comment: declared CoI - Nurithm Labs: Jyoti Mathur and Sharad Kumar profit beneficiaries, Vikas Chouhan employee. IRB AIIMS New Delhi. Funding: None (declared).
D1	Police et al [70]	Purposive (explicitly stated)	Yes	Single	No	No	NR	NR	5 classes (acne, tinea corporis, eczema, psoriasis, vitiligo); tinea corporis n = 8; single image/lesion (Samsung S23, natural light, 15-20 cm, no flash). Comment: app versions NR - limited temporal reproducibility. CoI: none declared.
D1	Yodrabum et al [59]	Mixed retrospective + prospective	Partial	Multi-source	No	No	NR	NR	Mixed dataset: Siriraj archive (derm. KOH+) + public DermNet (no mycological confirmation) + new collection (exclusively psoriasis). Anatomical exclusions: face/neck/inguinal lesions (Thai Personal Data Protection Act). IRB Siriraj, Mahidol Univ. (MU-MOU COA no. 073/2023).
D1	Zhu et al [71]	Retrospective	Yes	Single	No	No	NR	NR	OM: KOH (septate hyphae) and/or derm. culture and/or PAS+ with invasive hyphae. Controls: KOH+ culture-negative + clinical/histo criteria. Comment: selective (not systematic) PAS histology. Validation website: http://203.195.168.218/nail. IRB Sun Yat-sen Memorial (SYSEC-KY-KS-2021-113). Funding: NSFC Grant 81671992. [!] Dataset overlap with Kong2025.
D2	Berger et al [54]	Prospective consecutive	NR	Single	No	No	NR	NR	50 nail clippings (abnormal or possibly infected nails); 15/50 unusable (poor image quality - technical failure rate 30%). Comment: IRB NR; observers = medical students (not experts). Pre-DL technology (PAPNET/NNS); oldest study in the corpus.
D2	Decroos et al [49]	Retrospective	Yes	Single	No	No	NR	NR	Clinical suspicion of onychomycosis; nail biopsy; mean age 54 years (18-89); 104 M / 95 F; 169/199 toenail. Comment: random retrospective selection 2018-2020; OM prevalence 51% (101/199). IRB Ärztekammer Hamburg, no. WF-178/20. High CoI: 3 authors Mindpeak GmbH (proprietary software); declared "no conflicts" (incomplete).
D2	Gagna et al [72]	Retrospective	Partial	Single	No	No	NR	NR	14 patients with dermatophytoses (all T. rubrum); ages 6-24 years (mean 16.6); 3-mm punch biopsies supplied by Ideal Labs (Wayne, NJ). Comment: IRB not explicitly declared (specimens from an external provider). Exclusively pediatric/young population.
D2	Gao et al [73]	Prospective consecutive	Yes	Single	No	No	NR	NR	Dermatology patients with suspected fungal infection; age 15-78 years (mean 34.02); 156 M / 136 F. Comment: IRB undeclared; N = 292 patients (156 M + 136 F confirm distinct individuals). Proprietary Wangsheng stain; specific optical equipment - limited transferability. Co-author Zhang @ Guangzhou Wangsheng Intelligent Technology (maker of the evaluated stain) - Moderate CoI; declared "no conflicts" (incomplete).
D2	He et al [74]	Prospective consecutive	Yes	Single	No	No	NR	NR	Suspected SFI; no age/sex restrictions; mean 49 ± 19.38 years (2-87); M:F = 2.35:1; broad spectrum (tinea cruris 21.2%, corporis 15.4%, pedis 13.7%, onychomycosis 12.9%, seborrheic dermatitis 9.5%, Malassezia folliculitis 7.1%, PV 5.8%, etc.). Exclusions: topical/systemic antifungals in the previous 2 weeks. IRB Shanghai Skin Disease Hospital. CoI not explicitly declared.
D2	Hulse et al [75]	Prospective consecutive	Yes	Single	Yes - OSF osf.io/p8cnv (before participant accrual)	No	NR (R not published)	Yes - Mendeley Data 10.17632/53ssrx3cr9.1	Included: skin biopsies with PAS ordered for superficial fungal infection. Excluded: PAS for other indications; nail specimens (3 excluded post hoc). Preregistered protocol: OSF osf.io/p8cnv. The only preregistered study in the corpus. Partial D4 candidate (decisional output guides whether to order additional PAS).
D2	Jansen et al [50]	Retrospective	NR	Multi (4 labs, 3 scanners)	No	No	NR	NR	Routine cases from 4 dermatohistopathology laboratories (Germany + Austria); selection based on routine clinical diagnosis; PAS staining (different protocols per laboratory). IRB Univ.

Domain	Study	Patient selection	Demographic data	Centers	Protocol registration	External validation	Public code	Public data	Incl./excl. criteria + Comments (CoI, funding, IRB)
									Duisburg-Essen, no. 20-9196-BO; consent waived (retrospective, anonymous). High CoI: 3 authors Aignostics GmbH; declared "no conflict" (incomplete). Real-patient N NR (664 WSI; ~1 WSI/patient implied).
D2	Köberle et al [22]	Retrospective	NR	Single	No	No	Yes	NR	Patients with suspected pityriasis versicolor; specimens: torso (n = 18), face (n = 3), upper arm (n = 1); native tape strip without KOH. IRB: not required (BayKrG art. 27 §4 - routine data). Public code: GitHub ssitaru/malassezia-classifier.
D2	Koo et al [76]	Retrospective	Partial	Single	No	No	No	Yes	Patients with suspected fungal infection (skin + nails); Veterans Health Service Medical Center Seoul; Jan-Dec 2019; 18 positive (10 skin/nail at 40x + 8 at 100x) + 20 negative = 38 distinct patients. IRB Veterans Health Service Medical Center, no. 2020-02-013-001; consent waived. Public dataset FigShare: doi:10.6084/m9.figshare.14678514.v1. FigShare deposit contains images and label (txt) files only, no code; "public code" corrected from Yes to No.
D2	Mäder et al [55]	Prospective consecutive	NR	Single	No	No	NR	NR	Infected skin specimens from clinical cases (university hospital) + specimens from healthy subjects; MykoColor staining; 415 total images evaluated. IRB NR; patient N undeclared. Oldest D2 study (2015); pre-DL technology. Funding: LOEWE KMU-Verbundvorhaben (HA no. 294/11-39). Maximal CoI: co-author Quiskamp employed by Helmut Hund GmbH (maker of the evaluated imaging system); declared "no conflict" (incomplete).
D2	Rajitha et al [77]	Retrospective	NR	Single	No	No	NR	NR	Patients with dermatophytosis (skin scrapings); DVL clinic, Kasturba Medical College Manipal; dataset: 2626 patches (1363 fungal + 1263 non-fungal); KOH bright-field 40x. IRB: IEC no. 500/2020. Comment: dataset overlap with Rajitha2025 and Rajitha2025a.
D2	Rajitha et al [78]	Retrospective	NR	Single	No	No	No (explicitly stated unavailable)	NR	Patients with dermatophytosis (KOH positive); tertiary dermatology clinic, Kasturba Medical College Manipal; 256x256 px patches from KOH images at 10x and 40x. IRB IEC no. 500/2020. Dataset overlap with Rajitha2022/2025a.
D2	Rajitha et al [79]	Retrospective	NR	Single	No	No	NR	NR	Patients with confirmed dermatophytosis; tertiary dermatology clinic, Kasturba Medical College Manipal; 378 KOH images (skin scrapings); low/moderate/high grading. IRB IEC no. 500/2020. APC covered by Manipal Academy (Open Access). Dataset overlap with Rajitha2022/2025.
D2	Rappoport et al [80]	Retrospective	NR	Single	No	No	NR	NR	22 slides (7 PAS + 15 GMS) from a 2014-2023 archive; digitally scanned (Philips IntelliSite / Ventana DP200, x40); quality control (artifact exclusion). Nails and mucosae excluded. IRB Sheba Medical Center (no. 9801-22-SMC). Maximal CoI: 3 authors founders/employees of DeePathology Ltd. - Jacob Gildenblat, Ofir Hadar, Chen Sagiv; declared "None" (incomplete). POC-qualified study. Funding: None (declared). Reclassified: D1->D2.
D2	Ren et al [81]	Prospective consecutive	NR	Single	No	No	No (unavailable)	On request with EC approval	Clinical superficial-fungi specimens (skin); Huashan Hospital, Fudan University, Shanghai; collection Feb 2023-Feb 2024. IRB KY2023-057, Huashan Hospital. High CoI: Tan, Chen, Chen = SODA Data Technology (3 co-authors); Xu, Zhang = 3D Medicines Inc (2 co-authors). Annotation in APTime software (SODA - co-author company).
D2	Yilmaz et al [48]	Retrospective	Partial	Single	No	No	NR	NR	5 patients with distal lateral onychomycosis (KOH positive) + 5 control patients; Haydarpaşa Numune Training and Research Hospital, Istanbul; grayscale KOH images 40x. IRB HNEAH-KAEK 2021/209. Comment: circular reference.
D3	Anton et al [60]	Prospective consecutive	NR	Single (INOVIE network)	No	No	NR	NR	Clinical specimens: scrapings, nails, hair, swabs (suspected dermatophytosis); INOVIE network (Toulouse, Montpellier). Comment: training set partly on pure isolates and spiked specimens (~2/3 non-clinical). IRB: MR-002 compliance declaration/CNIL no. 2059942; Art. 54 French law. Maximal CoI: authors = founders/employees of Dendris SAS.
D3	Ivagnes et al [62]	Retrospective	NR	Multi (3: Rome, Vicenza, Delhi)	No	No	NR	NR	Patients with symptomatic dermatophytosis (erythema, pruritus, annular lesions); isolates from the T. mentagrophytes complex; 3 centers. IRB: Not applicable (declared) - a questionable declaration for real human clinical specimens. Moderate CoI: co-author Arroyo = employee of Clover Bioanalytical Software. Funding: EU Next Generation EU-MUR PNRR (PE00000007, INF-ACT).
D3	Tirard-Collet et al [61]	Retrospective	NR	Single	No	Partial (institutionally manufacturer-independent evaluation, single-center)	NR	NR	Suspected superficial fungal infection; Hospices Civils de Lyon (Hôpital Croix Rousse); 85 specimens; 31 Jul 2023-26 Jan 2024. Technical failure rate 5.9% (5/85 invalid PCR). IRB: waiver. Low CoI: kit supplied by Dendris, with no participation in design/interpretation. Co-author C. Miossec = author of the 2026 anchor reference. [Single study spanning both D2 and D3 in the domain partition - classified as D3 per molecular input (PCR + microarray)]
D3	Todd et al [63]	Retrospective (public-health surveillance)	Yes	Single	No	No	Yes (File S1 JCM)	Yes (GenBank PX687495-PX687856; NCBI SRA PRJNA1046065)	Patients with dermatophytosis caused by Ti, TmVII, or TmII*; NYC and neighboring counties; May 2022-Dec 2024; ages 4-79 years (Ti); 188 distinct patients. IRB: exempt (NYS DOH public-health surveillance); consent waived (de-identified data). CDC funding (public). No declared conflict. Closest D4 candidate: implicit genotype -> AFST resistance profile -> therapeutic-decision correlation.

Abbreviations (Tables S1-S3).

Acc, accuracy; AFST, antifungal susceptibility testing; ANN, artificial neural network; AUC, area under the ROC curve; CI, confidence interval; CNN, convolutional neural network; CoI, conflict of interest; COFRAC, French Committee for Accreditation; CV, cross-validation; Dice, Dice similarity coefficient; DTA, diagnostic test accuracy; DVL, Dermatology, Venereology and Leprosy; DWT, discrete wavelet transform; EDT, ensemble of decision trees; F1, F1 score; FF-NN, feed-forward neural network; FFPE, formalin-fixed paraffin-embedded; FMIA, fluorescence microscopy image analysis; FoV, field of view; GMS, Grocott methenamine silver; H&E, haematoxylin and eosin; HOMER, hierarchy of multilabel classifier; HSV, hue-saturation-value colour space; IHC, immunohistochemistry; IOU, intersection over union; IRB, institutional review board; ITS, internal transcribed spacer; KNN, k-nearest neighbours; KOH, potassium hydroxide; LightGBM (LGBM), light gradient-boosting machine; LOOCV, leave-one-out cross-validation; mAP, mean average precision; MALDI-TOF MS, matrix-assisted laser desorption/ionization time-of-flight mass spectrometry; MCC, Matthews correlation coefficient; MIC, minimum inhibitory concentration; ML, machine learning; NGS, next-generation sequencing; NPV, negative predictive value; NR, not reported; OM, onychomycosis; OSF, Open Science Framework; PAS, periodic acid-Schiff; PCR, polymerase chain reaction; PLS-DA, partial least-squares discriminant analysis; POC, proof of concept; PV, pityriasis versicolor; R-CNN, region-based convolutional neural network; ResNet, residual network; RF, random forest; ROC, receiver operating characteristic; RT-PCR, real-time PCR; Se, sensitivity; SE, squeeze-and-excitation; SFI, superficial fungal infection; SHAP, Shapley additive explanations; Sp, specificity; SRA, Sequence Read Archive; SVM, support vector machine; Ti, Trichophyton indotineae; TiTmSC, Trichophyton indotineae / Trichophyton mentagrophytes species complex; Tm, Trichophyton mentagrophytes; TL, transfer learning; UV, ultraviolet; VGG, Visual Geometry Group network; ViT, Vision Transformer; WSI, whole-slide imaging; XAI, explainable AI; YOLO, You Only Look Once; kappa, Cohen or Fleiss kappa.

Domains: D1, pre-analytical clinician-facing image processing; D2, analytical microscopy or histopathology; D3, analytical molecular techniques; D4, integrative post-analytical (empty). Genus and species names are italicised per journal style. Product and model names (for example DendrisCHIP, DendriSOFT, DendrisKIT, DermaAId, MSDAS, SwinIR, U2-Net) are given as in the source studies.
