# Supplementary material for: Artificial Intelligence in the Diagnosis of Superficial Mycoses: A Scoping Review
Source: J Fungi (Basel). 2026 Sep 11;12(9):684. doi: 10.3390/jof12090684 (PMC13608722; doi:10.3390/jof12090684)
Supplement: Supplementary file 1 [file jof-12-00684-s001.zip › Supplementary_Table_S5_CoI.pdf]

Supplementary Table S5. Conflicts of interest.

Conflicts of interest are reported descriptively. No validated risk-of-bias instrument exists for conflicts of interest, so no risk grade is assigned. Where an article declares "no conflict" but a tie to the evaluated tool was identified, the declaration is marked as not mentioning that tie.

Domains: D1 (studies 1-13), D2 (14-29), D3 (30-33). Of the 33 studies, 4 carry no conflict-of-interest statement (Idress, Kong, Miura, Berger), and the remainder either declare none or disclose the relationships shown.

Study	Declared conflict of interest	Industrial tie to the evaluated tool / reagent	Funding / IRB
Duzayak2023	Declares no conflict of interest.	None identified.	Funding NR; ethics approval Sakarya Univ. (E.13973, 06/11/2019).
Han2018c	Declares no conflict; discloses co-author W. Lim (SK Telecom).	None to the evaluated tool (SK Telecom is not the tool maker).	IRB Asan Medical Center (S2016-2209-0001; 2017-0087).
Idress2025	No conflict-of-interest statement in the article (conference paper).	None identified.	IRB not explicitly stated.
Julanon2025	Declares no conflict of interest.	None identified (public GitHub code).	IRB HE654011, Khon Kaen Univ.
Kim2020	Declares no conflict of interest.	None (industrial). Note: co-author S.S. Han created the algorithm evaluated here - a self-evaluation relevant to independence, not an industrial tie.	IRB Asan Medical Center (2018-1368).
Kong2025	No conflict-of-interest statement in the article.	None identified.	Hospital ethics committee approval.
Mehta2024	Declares "Conflict of interest: None".	None (no author affiliated with Nurithm; developers named only in Acknowledgments). Note: shares senior author S. Gupta with Pangti2021, whose tool is evaluated here (independence caveat).	AIIMS New Delhi Ethics Committee.
Miura2024	No conflict-of-interest statement in the article.	None identified.	IRB Akita Univ. (#2925, 27 Dec 2022).
Nigat2023	Declares no conflict of interest.	None identified.	IRB Jimma Univ. Institute of Technology.
Pangti2021	Discloses ties to Nurithm Labs (developer of the evaluated DermaAId tool).	Two authors (J. Mathur, S. Kumar) profit beneficiaries and one (V. Chouhan) employee of Nurithm Labs, which develops the evaluated DermaAId tool.	Funding: None (declared); IRB AIIMS New Delhi.
Police2025	Declares no conflict of interest.	None (evaluates 3 commercial apps; no author affiliated with the vendors).	Funding/IRB not stated.
Yodrabum2025	Declares no conflict of interest.	None identified.	IRB Siriraj, Mahidol Univ. (MU-MOU COA 073/2023).
Zhu2022	Declares no conflict of interest.	None identified.	Funding NSFC 81671992; IRB Sun Yat-sen Memorial (SYSEC-KY-KS-2021-113).
Berger2007	No competing-interest statement in the article (2007 letter).	None identified.	IRB NR.
Decroos2021	Declares "no conflicts" (does not mention the tie at right).	Three authors affiliated with Mindpeak GmbH; the study uses Mindpeak proprietary software.	IRB Ärztekammer Hamburg (WF-178/20).
Gagna2024	Declares no conflict of interest.	None (HALO AI accessed via an external core facility; specimens supplied by Ideal Labs).	IRB not declared; commercial specimens (Ideal Labs, Wayne, NJ).
Gao2021	Declares "no conflicts" (does not mention the tie at right).	Co-author Zhang affiliated with Guangzhou Wangsheng Intelligent Technology, maker of the evaluated proprietary Wangsheng stain.	IRB undeclared.
He2025	Declares no competing interests.	None (FMIA is a Chongqing Tianhai product, but no author is affiliated with the maker).	Funding NSFC (8217121063); IRB Shanghai Skin Disease Hospital.
Hulse2026	No explicit statement located; academic authors, no industrial affiliation.	None identified.	Preregistered on OSF (osf.io/p8cnv).
Jansen2022	Declares "no conflict" (does not mention the tie at right).	Three authors affiliated with Aignostics GmbH.	IRB Univ. Duisburg-Essen (20-9196-BO); PJ funded by UMEA.
Koberle2024	Declares no conflict of interest.	None (public code on GitHub).	IRB not required (BayKrG art. 27 §4).
Koo2021	Declares no competing interests.	None identified.	IRB Veterans Health Service Medical Center (2020-02-013-001).
Mäder2015	Declares "no conflict" (does not mention the tie at right).	Co-author Quiskamp employed by Helmut Hund GmbH, maker of the evaluated imaging / fluorescence system.	Funding LOEWE KMU-Verbundvorhaben (HA 294/11-39); IRB NR.
Rajitha2022	Declares no conflict of interest.	None identified.	IRB IEC 500/2020.
Rajitha2025	Declares no conflict of interest.	None identified.	IRB IEC 500/2020.
Rajitha2025a	Declares no conflict of interest.	None identified.	IRB IEC 500/2020; APC covered by Manipal Academy.
Rappoport2024	Declares "None" (does not mention the tie at right).	Three authors (J. Gildenblat, O. Hadar, C. Sagiv) founders / employees of DeePathology Ltd., maker of the evaluated DeePathology STUDIO.	Funding: None (declared); IRB Sheba Medical Center (9801-22-SMC).
Ren2025	Discloses employment of several co-authors (SODA Data Technology; 3D Medicines).	Three authors employed by SODA Data Technology (whose APTime software was used for annotation) and two by 3D Medicines Inc.	IRB Huashan Hospital (KY2023-057).
Yilmaz2022	Declares no conflict of interest.	None identified.	IRB HNEAH-KAEK 2021/209.
Anton2023	Discloses: 6 authors employees, 2 founders of Dendris SAS.	Six authors employed by, and two founders of, Dendris SAS, which produces the evaluated DendrisCHIP DP.	No external funding; MR-002 compliance / CNIL 2059942 (ethics waived).
Ivagnes2025	Discloses co-author affiliation (Clover Bioanalytical Software).	Co-author Arroyo employed by Clover Bioanalytical Software S.L., whose MSDAS software was used.	Funding EU Next Generation EU-MUR PNRR; IRB "Not applicable" (questionable for 56 human isolates).
TirardCollet2025	Discloses that Dendris supplied the device without any role in design, execution, interpretation, or writing.	DendrisKIT supplied by Dendris (no design role); co-author C. Miossec is author of the 2026 anchor reference.	No external funding; IRB waiver.
Todd2026	Declares no conflict of interest.	None (public-health surveillance laboratory).	CDC funding (public); IRB exempt (NYS DOH surveillance).
