# Supplementary material for: Artificial Intelligence in the Diagnosis of Superficial Mycoses: A Scoping Review
Source: J Fungi (Basel). 2026 Sep 11;12(9):684. doi: 10.3390/jof12090684 (PMC13608722; doi:10.3390/jof12090684)
Supplement: Supplementary file 1 [file jof-12-00684-s001.zip › Table_S4_search_strategies.pdf]

Table S4. Search queries.

Each equation is given in full in a single cell so that it can be copied and run directly. Within each block, terms are combined with OR; blocks are combined with AND. The asterisk (*) denotes truncation. All searches were executed on 7 May 2026, which defines the corpus cutoff; no restriction on publication year was applied.

Database	Complete search equation (copy and run)	Limits / notes
**PubMed / MEDLINE**<br>https://pubmed.ncbi.nlm.nih.gov/	`("Artificial Intelligence"[MeSH] OR "Deep Learning"[MeSH] OR "Machine Learning"[MeSH] OR "Neural Networks, Computer"[MeSH] OR "convolutional neural network*"[tiab] OR "deep learning"[tiab] OR "machine learning"[tiab] OR "computer-aided diagnos*"[tiab] OR "computer assisted diagnos*"[tiab] OR "image analysis"[tiab] OR "vision transformer*"[tiab]) AND ("Tinea"[MeSH] OR "Onychomycosis"[MeSH] OR "Dermatomycoses"[MeSH] OR "Candidiasis, Cutaneous"[MeSH] OR "Tinea Versicolor"[MeSH] OR dermatophytos*[tiab] OR "superficial myco*"[tiab] OR "fungal skin infection*"[tiab] OR "nail myco*"[tiab] OR onychomycos*[tiab] OR tinea[tiab] OR "pityriasis versicolor"[tiab] OR "cutaneous candid*"[tiab]) AND ("Diagnosis"[MeSH] OR "Diagnostic Imaging"[MeSH] OR diagnos*[tiab] OR detect*[tiab] OR classif*[tiab] OR identif*[tiab])`	Humans; English; no date restriction. Three blocks (AI × superficial mycoses × diagnosis) combined with AND.
**Europe PMC**<br>https://europepmc.org/	`(TITLE_ABS:"artificial intelligence" OR TITLE_ABS:"deep learning" OR TITLE_ABS:"machine learning" OR TITLE_ABS:"convolutional neural network" OR TITLE_ABS:"neural network") AND (TITLE_ABS:onychomycosis OR TITLE_ABS:tinea OR TITLE_ABS:dermatophyt* OR TITLE_ABS:"pityriasis versicolor" OR TITLE_ABS:"cutaneous candidiasis" OR TITLE_ABS:"fungal skin")`	English; no date restriction. Restricted to title/abstract (TITLE_ABS:) because the full-text default mis-parsed the nested Boolean AND.
**arXiv**<br>https://arxiv.org/	`#1  (onychomycosis OR tinea OR "fungal skin") AND ("deep learning" OR "neural network")   [returned 0 records]`<br>`#2 onychomycosis   #3 tinea   #4 dermatophyte   #5 ringworm   #6 fungal skin infection   [successive single-term searches, manual screening]`	No date restriction. The combined Boolean query returned no records; disease names were then searched as successive single terms with manual screening.
**OpenAlex**<br>https://openalex.org/	`("artificial intelligence" OR "deep learning" OR "machine learning") AND (onychomycosis OR tinea OR dermatophyte* OR "pityriasis versicolor" OR "fungal skin")`	No date restriction. Full-text keyword search; document-type filters applied via the graphical interface.
**Dimensions**<br>https://app.dimensions.ai/	`("artificial intelligence" OR "deep learning" OR "machine learning" OR "convolutional neural network" OR "neural network" OR "AI-based" OR "AI-powered" OR "AI-assisted" OR "automated detection" OR "automated diagnosis" OR "automated grading" OR "automated classification" OR "computer-aided" OR "computer-assisted" OR "transfer learning" OR "vision transformer") AND (onychomycosis OR tinea OR dermatophyte* OR "pityriasis versicolor" OR "cutaneous candidiasis" OR "fungal skin" OR ringworm OR "skin mycosis")`	English; no date restriction. Run in "Title and Abstract" mode (Advanced Search). Publication type: Article, Review, Preprint, Chapter, Proceeding.
